# Defective DNA single-strand break repair is responsible for senescence and neoplastic escape of epithelial cells

**DOI:** 10.1038/ncomms10399

**Published:** 2016-01-29

**Authors:** Joe Nassour, Sébastien Martien, Nathalie Martin, Emeric Deruy, Elisa Tomellini, Nicolas Malaquin, Fatima Bouali, Laure Sabatier, Nicolas Wernert, Sébastien Pinte, Eric Gilson, Albin Pourtier, Olivier Pluquet, Corinne Abbadie

**Affiliations:** 1 Univ. Lille, CNRS, Institut Pasteur de Lille, UMR 8161 - M3T - Mechanisms of Tumorigenesis and Targeted Therapies, F-59000 Lille, France; 2 Commissariat à l'Energie Atomique (CEA), Laboratoire de Radiobiologie et Oncologie (LRO), 18 route du Panorama - BP6, 92265 Fontenay-aux-Roses 53011, France; 3 Institute of Pathology, University of Bonn, 53011 Bonn, Germany; 4 Institute for Research on Cancer and Aging, Nice (IRCAN), University of Nice Sophia Antipolis, CNRS, UMR7284, INSERM U108, Faculty of Medecine of Nice; CHU of Nice, Nice, France

## Abstract

The main characteristic of senescence is its stability which relies on the persistence of DNA damage. We show that unlike fibroblasts, senescent epithelial cells do not activate an ATM-or ATR-dependent DNA damage response (DDR), but accumulate oxidative-stress-induced DNA single-strand breaks (SSBs). These breaks remain unrepaired because of a decrease in PARP1 expression and activity. This leads to the formation of abnormally large and persistent XRCC1 foci that engage a signalling cascade involving the p38MAPK and leading to p16 upregulation and cell cycle arrest. Importantly, the default in SSB repair also leads to the emergence of post-senescent transformed and mutated precancerous cells. In human-aged skin, XRCC1 foci accumulate in the epidermal cells in correlation with a decline of PARP1, whereas DDR foci accumulate mainly in dermal fibroblasts. These findings point SSBs as a DNA damage encountered by epithelial cells with aging which could fuel the very first steps of carcinogenesis.

## Abstract (web summary)

It is recognized that cellular senescence is triggered by DNA damage as a protective mechanism against tumorigenesis. Here the authors show that DNA single-strand breaks of oxidative origin can induce a transient senescent state followed by the emergence of clonal transformed cells.

## Graphical abstract

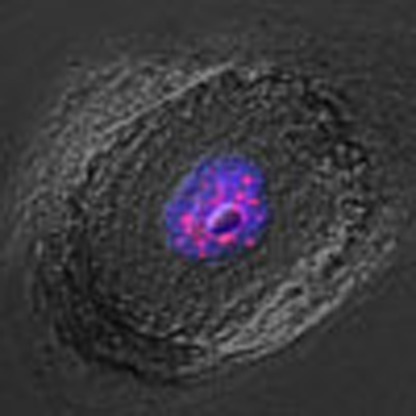

All normal human cells display a limited proliferative life span, at the end of which they enter a state known as senescence^1^. Cell senescence occurs *in vivo* as well as *in vitro*^2^,^3^,^4^ and is primarily characterized by a cell cycle arrest. The senescent phenotype also includes cell enlargement, increase in senescence-associated-*β*-galactosidase (SA-*β*-Gal) activity reflecting an expansion of the lysosomal compartment^2^,^5^, increase in autophagic activity^6^,^7^, altered chromatin organization^8^ and a specific inflammatory secretome^9^,^10^,^11^,^12^. Senescence is triggered by various cellular stresses including telomere erosion or dysfunction^13^, persistent DNA damage^14^, strong mitogenic signals^15^ and oxidative stress^16^. The robustness and irreversibility of the senescent cell cycle arrest and the presence of numerous senescent cells in pre-neoplastic lesions^17^,^18^,^19^,^20^ suggest that senescence is an onco-suppressive safeguard mechanism that opposes the development of malignant cancer. The underlying molecular mechanisms were established using mainly normal human fibroblasts in which it was shown that senescence is accompanied by telomere shortening and accumulation of irreparable DNA double-strand breaks (DSBs), which both induce a robust and persistent DNA damage response (DDR) orchestrated by the ATM/ATR kinases, and a sustained activation of the p53/p21(TP53/CDKN1A) pathway followed by permanent cell cycle arrest^13^,^14^,^15^. This is in accordance with the fact that sarcomas (fibroblast-originating tumours) are very rare in humans (<1% of total human cancers) with an incidence which does not vary with age. In contrast, this is in opposition with the fact that carcinomas (epithelial cell-originating tumours), which are the most prevalent cancers in humans, have an incidence which dramatically increases with advancing age. One presently proposed explanation for the increase in carcinoma incidence with age is the senescing of the fibroblasts whose secretome was shown to promote the tumoral development of already premalignant cells. However, this senescent secretome was found to have no transforming effects on normal cells^21^. This suggests that the very first steps of carcinogenesis at advanced age are activated by some other unknown mechanisms. These mechanisms could potentially be intrinsic to epithelial cell senescence. Indeed, we and others have provided evidence that *in vitro* cultured human keratinocytes and mammary epithelial cells spontaneously overcome senescence and give rise to transformed or genetically unstable cells^22^,^23^,^24^. We have shown that the post-senescent keratinocytes are generated from a few fully senescent mother cells^24^, suggesting that keratinocytes undergo some modification during senescence, including potentially mutagenic DNA damage, that predispose them for neoplastic transformation.

In the keratinocyte model, we have established that senescence is induced, at least in part, by a NF-κB→MnSOD→H_2_O_2_ pro-oxidant pathway^25^. This pathway has deleterious consequences and leads to the final death of most senescent keratinocytes by autophagic programmed cell death^24^,^26^. Paradoxically, this pathway also has the capacity to promote post-senescence neoplastic evasion, in correlation to its ability to induce oxidative DNA damage^24^. The most abundant oxidative-stress-induced DNA lesions are single-strand breaks (SSBs). They arise either directly via the fragmentation by oxidation of the phosphate-deoxyribose backbone or indirectly during the repair of oxidized bases by the mechanism of base excision repair (BER), at the step of the enzymatic excision of the damaged base^27^,^28^. Mammalian cells have evolved SSB repair (SSBR) pathways and are assumed to be relativity tolerant to these oxidative DNA damages which are not lethal. However, oxidized bases are reported as highly mutagenic^29^, and unrepaired SSBs can become clastogenic through the generation of one-ended DSBs resulting from collapsed replication forks^27^. Hence, oxidative DNA damage could be a discreet but potent inducer of tumorigenesis.

We report here that, in contrast to fibroblasts which undergo an irreversible senescence associated with an accumulation of persistent telomeric and non-telomeric DDR foci, epithelial cells accumulate persistent SSBR foci which drive the establishment of a transient senescent plateau followed by emergence of clones of transformed and mutated cells.

## Results

### Senescent NHEKs spontaneously give rise to neoplastic cells

**Figure 1:**
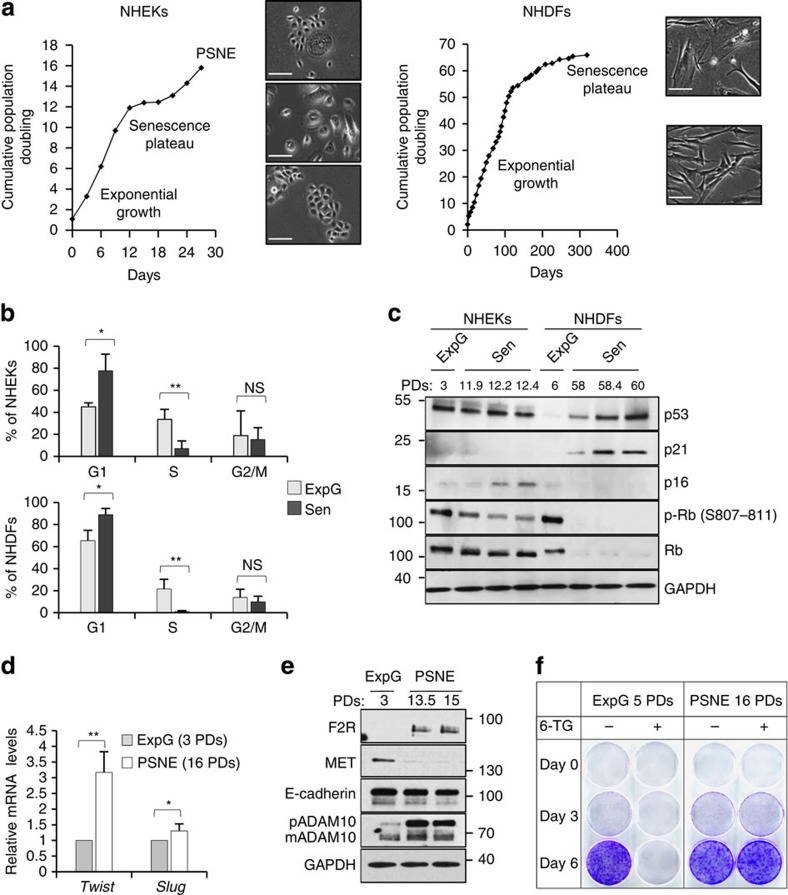
Growth curve and characteristics of NHEKs and NHDFs. (**a**) Growth curve of NHEKs (left panel) and NHDFs (right panel; donor 1MC) with representative micrographs of each growth phase; Scale bar, 50 μm. (**b**) Cell cycle distribution of exponentially growing and senescent NHEKs and NHDFs. The bar chart represents the means±s.d. of the means of three experiments performed with three different NHEKs-NHDFs couples: K1MC (ExpG: 3 PDs—Sen: 12.3 PDs), F1MC (ExpG: 11 PDs—Sen: 58 PDs), K1320 (ExpG: 3.5 PDs—Sen: 10 PDs), F1320 (ExpG: 11 PDs—Sen: 39 PDs), K67FA1 (ExpG: 2 PDs—Sen: 13 PDs) and F67FA1 (ExpG: 10.5 PDs—Sen: 42 PDs). (**c**) Western blot analysis of p53, p21, p16, Rb phosphorylated on serine 807 and 811 (p-Rb (S807–811)), Rb and GAPDH (loading control) levels in total cell extracts of NHEKs and NHDFs (donor 1MC). (**d**) Reverse-transcription quantitative real-time PCR (RT–qPCR) analysis of twist and slug transcripts in exponentially growing and PSNE NHEKs (donor 67FA1). Results are mean±s.d. of triplicates. Similar results were obtained with donor 1MC. (**e**) Western blot analysis of F2R, MET, E-cadherin, ADAM10 (pro-ADAM10 and mature-ADAM10) and GAPDH (loading control) levels in total NHEKs extracts (donor 1MC). (**f**) HPRT assays performed in NHEKs (donor 1MC). The quantification of the results is given in Supplementary Fig. 14. ExpG, exponentially growing cells; Sen, cells at the senescence plateau. The exact PDs at which cells were taken is indicated.

To find out senescence-associated molecular mechanisms involved in tumor initiation, we took advantage of comparing two cell types differing in their ability to spontaneously evade senescence and generate neoplastic cells: normal human dermal fibroblasts (NHDFs) and normal human epidermal keratinocytes (NHEKs). Pairs of isogenic NHDFs and NHEKs (listed in Supplementary Table 1) were used in all experiments to avoid any difference due to individual polymorphism. Cells were grown at the atmospheric O_2_ tension which is nearly normoxic for epidermis and partially hyperoxic for dermis^30^. NHDFs reach senescence after an exponential growth phase lasting 30–70 population doublings (PDs; Fig. 1a and Supplementary Fig. 1). The senescence plateau is associated with cell-size enlargement (Fig. 1a), cell flattening (Fig. 1a), increase in SA-*β*-Gal activity (Supplementary Fig. 2A), cell cycle arrest in G1 (Fig. 1b, Supplementary Fig. 2A,B) and activation of the p53/p21 pathway (Fig. 1c). In comparison, NHEKs enter senescence after only 10–15 PDs (Fig. 1a and Supplementary Fig. 1). They display canonical senescence markers, including flattening and enlargement (Fig. 1a), increase in SA-*β*-Gal activity (Supplementary Fig. 2A) and cell cycle arrest in G1 (Fig. 1b, Supplementary Fig. 2A,B). However, their cell cycle arrest is not associated with a detectable activation of p53/p21 but instead of p16/Rb (CDKN2A/RB1; Fig. 1c, Supplementary Fig. 3). Importantly, in contrast to NHDFs, their senescence plateau is transient. While most senescent NHEKs die by autophagic cell death^7^,^26^, about 1 out of every 10,000 undergoes an unusual budding mitosis which generates clones of standard-sized cells that have lost the SA-*β*-Gal staining, re-proliferate and rapidly supplant senescent cells (Fig. 1a, Supplementary Fig. 4 and ref. 24). Filiation-tracing assays and videomicroscopies have proven that these cells do come from the division of fully senescent mother cells^24^. A transcriptomic analysis revealed that these cells display a gene signature of pre-transformation^31^. Moreover, they exhibit a partial epithelial-mesenchymal transition (EMT) with an increase in expression of TWIST and SLUG (Fig. 1d), a decrease in expression of MET, a slight decrease in expression of E-cadherin (CDH1) and an increase in expression of F2R (TR, PAR1) and ADAM10 (Fig. 1e). This partial EMT drastically exacerbates when cells are exposed to the secretome of senescent NHDFs, with acquisition of a fibroblastoid morphology and migratory capacities^32^. Remarkably, post-senescent cells are mutated as assessed by hypoxanthine-guanine phosphoribosyltransferase (HPRT) assays (Fig. 1f) and were shown to form disseminated skin hyperplasia and small carcinomas when xenografted into nude mice^24^. Due to all these properties of transformation and tumorigenicity, we called these cells post-senescent neoplastic emergent (PSNE) cells. PSNE clones were systemically obtained with each donor regardless of sex, age or race (Supplementary Fig. 1 and refs 24, 26, 31, 33). Moreover, rare small islets of keratinocytes expressing F2R were detected in aged skin^32^.

### Senescent NHEKs do not activate the DDR pathway

**Figure 2:**
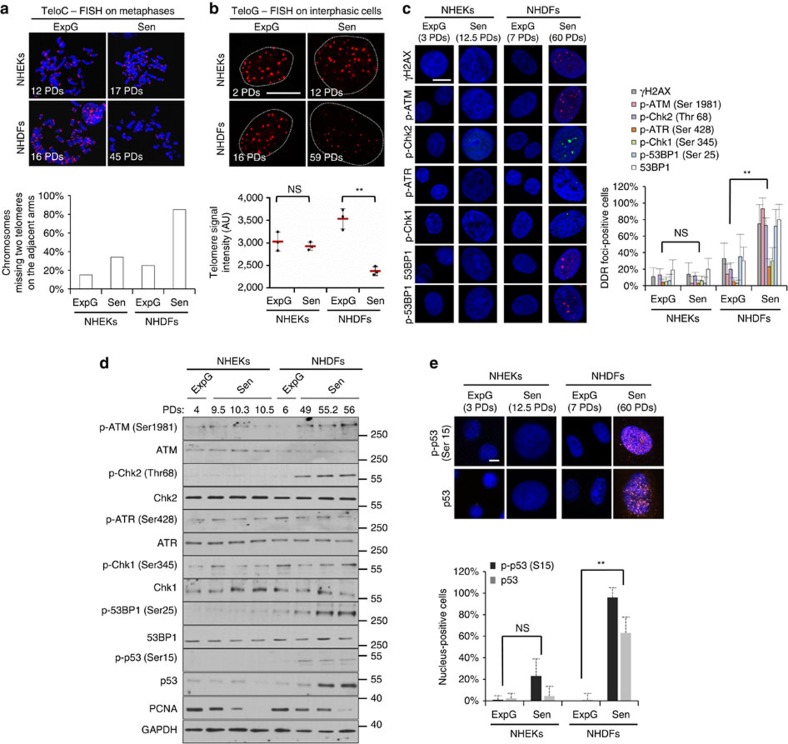
Senescent NHEKs do not experience massive telomere shortening nor activate the DDR pathway. (**a**) Telo-FISH on metaphase chromosome spreads of NHEKs and NHDFs (donor 2F19). Upper panel: representative Telo-FISH images. Lower panel: quantification of telomeres loss. The given results are the mean of counts performed on 45–58 metaphases for each case. (**b**) Telo-FISH on interphasic cells. Upper panel: representative confocal microscopy images for the 1MC donor. Scale bar, 20 μm. Lower panel: quantification of the fluorescence intensity obtained with three different NHEKs-NHDFs couples (1MC, 1320 and 67FA1). Scatter dot plots indicate the means±s.d. of the means of the three experiments. (**c**) Analysis by immunofluorescence of the activation of the DDR pathway in NHEKs and NHDFs (donor 1MC). Left: representative ApoTome microscopy images of DDR foci. Scale bar, 20 μm. Right: quantification of the number of cells displaying at least three foci of γH2AX, p-ATM, p-Chk2 or 53BP1, or at least one foci of p-ATR or p-Chk1. DDR foci-positive cells were automatically counted with ImageJ in 10 independent microscopic fields for a total of at least 200 cells for each case. The bar chart represents the mean±s.d. of each 10 counts. The results are representative of three independent experiments. (**d**) Western blot analysis of the activation of the DDR pathway in total cell extracts of NHEKs and NHDFs (donor 1MC). PCNA was used as proliferative index and GAPDH as loading control. (**e**) p53 and p–p53 (Ser15) immunofluorescences performed on NHEKs and NHDFs (donor 1MC). Upper panel: representative ApoTome microscopy images. Scale bar, 20 μm. Lower panel: quantification of cells displaying p53 and p–p53 (Ser15) nuclear staining. Cells were counted in five independent microscopic fields for a total of at least 100 cells for each case. The bar chart represents the mean±s.d. of each 10 counts. The results are representative of two independent experiments. ExpG. exponentially growing cells; Sen, cells at the senescence plateau. The exact PDs at which cells were taken is indicated.

Since one inducer of senescence is telomere shortening, we compared the telomere status of NHDFs and NHEKs by performing fluorescence *in situ* hybridization (FISH) on interphasic cells and metaphase spreads. As expected, we observed a drastic decrease in telomeric signal in NHDFs at senescence. In contrast, most of the chromosomes remained telomere-positive in senescent NHEKs (Fig. 2a,b), although the telomerase is not reactivated^24^. Therefore, most NHEKs still have a doubling potential when reaching the senescence plateau.

Since senescence was shown to be induced by a DDR signalization emanating from shortened telomeres but also from DSBs elsewhere in the genome^34^, we compared the activation of the DDR pathway in the two cell types. Almost all senescent NHDFs displayed 3–5 nuclear large foci of phosphorylated ATM, H2AX, CHEK2 and 53BP1 (Fig. 2c,d), and were positive for the activated form of p53 phosphorylated on serine 15 (Fig. 2d,e). To determine whether these DDR foci were telomere-induced foci, we performed a co-detection of 53BP1 and the telomeric protein TRF2. We found that some, but not all, 53BP1 foci were located at telomeres (Supplementary Fig. 5). In striking contrast to NHDFs, NHEKs did not activate the DDR pathway at senescence and most of them were negative for activated p53 (Fig. 2c–e).

### Senescent NHEKs have a dysfunctional SSBR pathway

**Figure 3:**
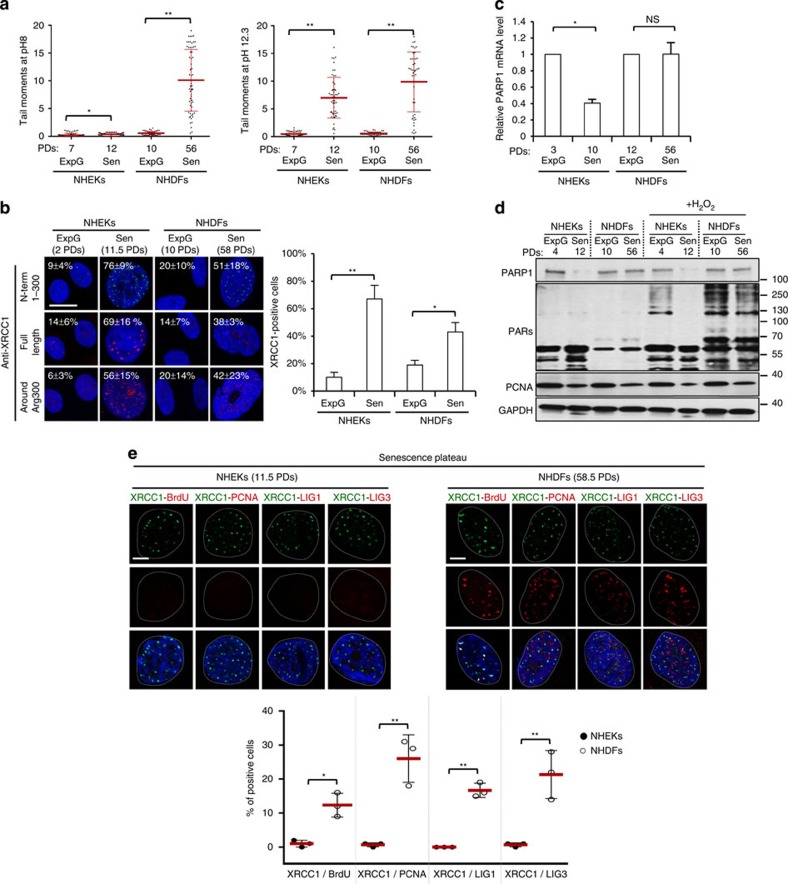
Senescent NHEKs display a decrease in PARP1 expression and activity and accumulate unrepaired SSBs. (**a**) Neutral (pH 8; left) and alkaline (pH 12.3; right) comet assays performed in tandem on exponentially growing and senescent NHEKs and NHDFs (donor 1MC). Microphotographs of the comets are shown in Supplementary Fig. 6A. Tail moments of 30–50 comet-positive cells were quantified. Scatter dot plots represent the mean±s.d. The results are representative of five independent experiments. (**b**) XRCC1 immunofluorescence performed on exponentially growing and senescent NHEKs and NHDFs (donor 1MC) using three different antibodies raised against different XRCC1 immunogens. The specificity of the antibodies is analysed in Supplementary Fig. 6B and C. Left: representative ApoTome microscopy images. Scale bar, 20 μm. Right: XRCC1 foci-positive cells were automatically counted with ImageJ in five independent microscopic fields for a total of at least 100 cells for each case. The mean±s.d. of the five counts is indicated as inserts. The bar chart represents the means±s.d. of the means obtained with the three antibodies. (**c**) Reverse-transcription quantitative real-time PCR (RT–qPCR) analysis of PARP1 transcripts (donor 1MC). Results are means±s.d. of triplicates. Similar results were obtained with the 67FA1 donor. (**d**) Western blot analysis of PARP1, PAR, PCNA (proliferative index) and GAPDH (loading control) levels in total cell extracts of exponentially growing and senescent NHEKs and NHDFs (donor 1 MC) treated or not with 100 μM H_2_O_2_ at 4 °C for 10 min and then placed at 37 °C for 5 min. The specificity of PARP1 and PAR antibodies is analysed in Supplementary Fig. 7B. (**e**) Double immunofluorescence detection of XRCC1 with BrdU, Ligase1, Ligase3 or PCNA. Upper panel: representative ApoTome microscopy images obtained with the 1MC donor. Scale bar, 10 μm. Similar results were obtained with the 1320 and 67FA1 donors. Lower panel: cells displaying double-positive foci were automatically counted with ImageJ in 10 fields for a total of >100 nuclei and the means were calculated. Scatter dot plots represents the mean±s.d. of the means of the three experiments performed with the three different donors. ExpG, exponentially growing cells; Sen, cells at the senescence plateau. The exact PDs at which cells were taken is indicated.

Since senescent NHEKs do not activate the DDR pathway, we wondered what could induce their cell cycle arrest. We examined whether they accumulate SSBs and activate the SSBR pathway. We quantified SSBs using tandem neutral (pH 8) and alkaline (pH 12.3) comet assays that are indicative of DSBs and of the sum of SSBs+DSBs respectively. The results were analysed by calculating the tail moments, which reflect the extent of DNA breaks per comet-positive cell. The tail moments at pH 8 increased at senescence only in NHDFs, whereas at pH 12.3 they increased in both NHEKs and NHDFs (Fig. 3a and Supplementary Fig. 6A), indicating that NHEKs accumulate at senescence only SSBs, whereas NHDFs accumulate both SSBs and DSBs.

The SSBR pathway involves first a poly(ADP) ribose-polymerase (PARP), primarily PARP1, which binds to the broken DNA. This binding enhances its poly(ADP) ribose (PAR) polymerization activity. The accumulated PARs serve the recruitment of XRCC1, a scaffold protein, which in turn recruits the downstream repair enzymes^35^,^36^. By immunofluorescence, we observed an increase in XRCC1 foci at senescence in both NHEKs and NHDFs, but more prominently in NHEKs (Fig. 3b; for the specificity of the antibodies, see Supplementary Fig. 6B,C). Since XRCC1 also functions during BER, we wanted to distinguish the XRCC1-containing SSBR foci from the XRCC1-containing BER foci. Double immunofluorescences against XRCC1 and hOGG1, the DNA glycosylase responsible for the excision of damaged bases^37^,^38^ show that most of both senescent NHEKs and NHDFs displayed XRCC1 foci but no hOGG1 foci (Supplementary Fig. 7A). Therefore, senescence is accompanied by an accumulation of direct SSBs and activation of the SSBR pathway, more prominently in NHEKs than in NHDFs.

To understand why NHEKs accumulate more SSBs than NHDFs, we investigated their repair capacities. We examined first the expression of PARP1. Its mRNA and protein levels dramatically decreased at senescence in NHEKs, whereas they remained nearly stagnant in senescent NHDFs (Fig. 3c,d and Supplementary Fig. 7C; Supplementary Fig. 7B for the specificity of the antibody). We further investigated PARP1 activity. Cells were treated with 100 μM H_2_O_2_, to induce numerous SSBs, and the production of PARs was analysed by western blot and immunofluorescence (see Supplementary Fig. 7B for the specificity of the antibody). The results show that exponentially growing versus senescent NHDFs respond to H_2_O_2_ by producing PARs nearly equally, whereas senescent NHEKs were almost completely unable to produce PARs (Fig. 3d and Supplementary Fig. 7C).

With diminished PARP1 expression and activity, senescent NHEKs should be unable to repair their SSBs. To test this assumption, we processed cells for BrdU incorporation to mark the foci undergoing repair. Senescent NHDFs displayed BrdU foci that co-localized with XRCC1 foci, whereas senescent NHEKs did not show any BrdU foci despite the presence of numerous XRCC1 foci (Fig. 3e). We then analysed the recruitment of proliferating cell nuclear antigen (PCNA), ligases 1 and 3. In about 20% of senescent NHDFs, PCNA and ligases 1 and 3 were found to be recruited at XRCC1 foci. In contrast, senescent NHEKs did not show any PCNA, ligases 1 or 3 foci (Fig. 3e).

Taken together, these results provide evidence that the SSBR pathway of NHEKs is compromised at senescence by a decrease in PARP1 expression and activity, leading to accumulation of unrepaired SSBs. NHDFs also displays numerous SSBs at senescence, but with a functional SSBR pathway, these SSBs are continuously repaired.

### Senescent NHEKs display enlarged and persistent XRCC1 foci

**Figure 4:**
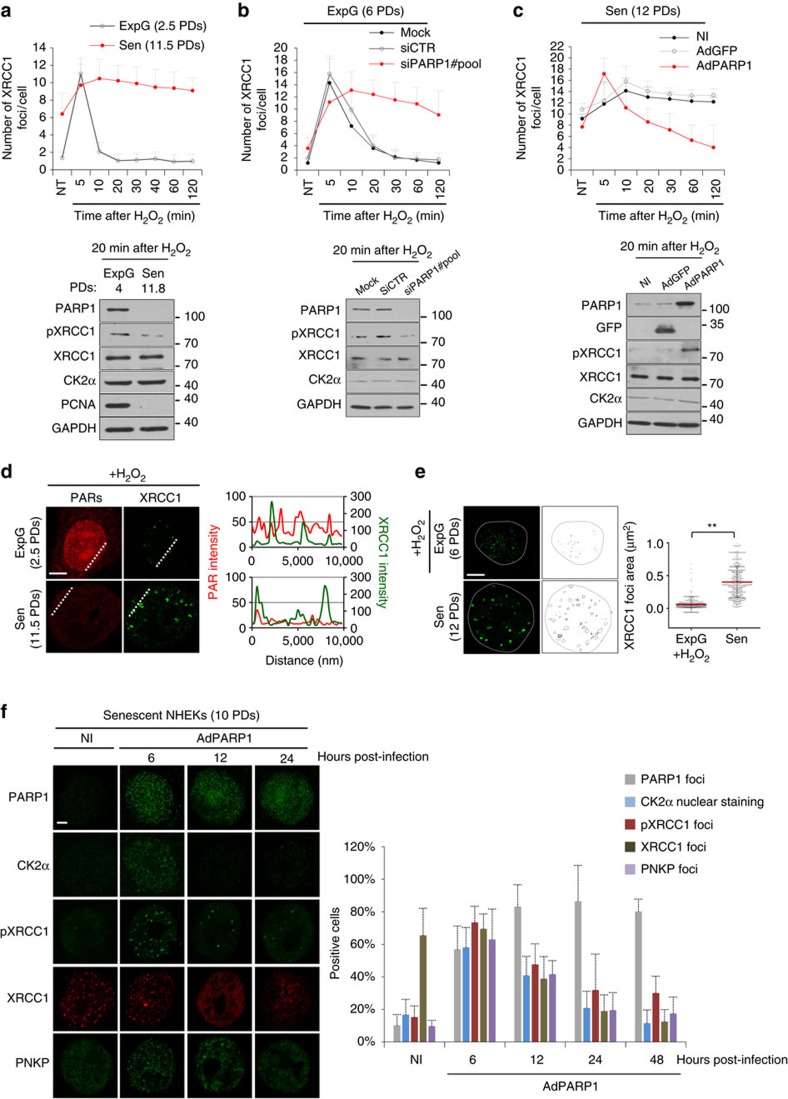
Distinctive features of XRCC1 foci at senescence in NHEKs. (**a**) Upper panel: follow-up of XRCC1 foci in exponentially growing and senescent NHEKs (donor 1MC) treated by 100 μM H_2_O_2_ at 4 °C for 10 min and then placed at 37 °C for 5 to 120 min. The number of foci per cell was counted in >50 cells. Each point represents the mean±s.d. Lower panel: exponentially growing and senescent NHEKs (donor 67FA1) were treated by 100 μM H_2_O_2_ at 4 °C for 10 min, placed at 37 °C for 20 min and analysed by western blot for PARP1, XRCC1, phosphorylated XRCC1 (S518/T519/T523), CK2α, PCNA (proliferative index) and GAPDH (loading control). (**b**) Exponentially growing NHEKs (donor 67FA1) were transfected or not with a pool of four siRNAs against PARP1 or a pool of four control siRNAs. Forty-eight hours after transfection, the same analyses as in **a** were performed. (**c**) Senescent NHEKs (donor 67FA1) were infected with adenoviral vector encoding PARP1 (AdPARP1), adenovirus encoding green fluorescence protein (AdGFP) or kept non-infected (NI). 6 h after infection, the same analyses as in **a** were performed. (**d**) Exponentially growing and senescent NHEKs (donor 1MC) were treated by 100 μM H_2_O_2_ at 4 °C for 10 min and then placed at 37 °C for 5 min. Left panels: representative confocal photomicrographs of PAR and XRCC1 foci. Scale bar, 10 μm. Right panels: measures of fluorescence intensity performed along the dotted lines. (**e**) Measure of XRCC1 foci area in H_2_O_2_-treated exponentially growing and non-treated senescent NHEKs. Left: representative confocal photomicrographs of XRCC1 foci. Scale bar, 10 μm. Right: area of at least 100 foci measured by ImageJ. Scatter dot plots represent the mean±s.d. (**f**) Senescent NHEKs (donor 67FA1) were infected with AdPARP1 or kept non-infected (NI) and fixed at 6, 12, 24 and 48 h post-infection. Left panel: representative photomicrographs of PARP1, CK2α, phosphorylated XRCC1, XRCC1 and PNKP immunostainings. Scale bar, 5 μm. Right panel: quantification of cells displaying PARP1 foci, CK2α nuclear staining, phosphorylated XRCC1 foci, total XRCC1 foci and PNKP foci. At least 100 cells were counted for each condition. Each point represents the mean±s.d. ExpG, exponentially growing cells; Sen, cells at the senescence plateau. The exact PDs at which cells were taken is indicated.

XRCC1 being recruited by binding to PARs, and PARP1 activity being decreased at senescence, we wondered how the XRCC1 foci form at senescence. When SSBs were induced by a H_2_O_2_ treatment, XRCC1 foci formed within 5 min in exponentially growing NHEKs, and with a delay of five additional minutes in senescent NHEKs. They resolved within 10 min in exponentially growing NHEKs, whereas in senescent NHEKs they persisted for >2 h (Fig. 4a). This suggested that the recruitment of XRCC1 at the breaks was, at senescence, slightly slowed down but still effective and, above all, that the dissociation of XRCC1 from the foci was almost abrogated. To establish whether this alteration in the dynamics of the foci was the consequence of the poor PARP1 expression at senescence, we decreased PARP1 expression in exponentially growing NHEKs using short interfering RNAs (siRNAs). This recapitulated both the delay in formation and the persistence (Fig. 4b). We conversely restored PARP1 expression in senescent NHEKs by infecting them with an adenoviral vector encoding PARP1. This restored the normal formation speed and greatly accelerated the resolution (Fig. 4c). These results suggested that a small quantity of PARs was (i) enough to recruit XRCC1, although at a reduced speed, but (ii) insufficient to release it. To further support the first assumption, we treated exponentially growing and senescent NHEKs with H_2_O_2_ and recorded by confocal microscopy the intensity of the PAR and XRCC1 staining at the foci. The PAR staining intensity in the foci at senescence was very faint compared with that in exponentially growing cells. Nevertheless, the XRCC1 staining intensity at the same foci was similar in exponentially growing and senescent NHEKs (Fig. 4d), showing that the poor PAR synthesis occurring at senescence is enough to normally recruit XRCC1. To further support the second assumption, we made three experiments. First, we compared the size of the XRCC1 foci at senescence to that of normal foci. Indeed, if the dissociation of XRCC1 is impaired, XRCC1 should accumulate and the foci should be abnormally large. While H_2_O_2_-treated exponentially growing NHEKs developed a narrow range of small foci, senescent NHEKs displayed a wider range of 4.7-fold larger foci (Fig. 4e). Second, we investigated the phosphorylation of XRCC1 by CK2α (CSNK2A1) which was shown to be required for the proper recruitment of PNKP and APTX, two enzymes involved in the restoration of the 3′- and 5′-termini and for the proper dissociation of XRCC1^39^,^40^,^41^ (See Supplementary Fig. 8A for the specificity of the antibodies). Interestingly, XRCC1 was less phosphorylated in senescent than in exponentially growing NHEKs (Fig. 4a). It was also less phosphorylated in exponentially growing NHEKs invalidated for PARP1 (Fig. 4b). Conversely, the phosphorylation was restored upon re-expression of PARP1 in senescent NHEKs (Fig. 4c,f). The restoration of phosphorylation occurred concomitantly with the translocation of CK2α in the nucleus and with the recruitment of PNKP at the foci, and preceded the release of XRCC1 from the foci towards the rest of the chromatin (Fig. 4f, Supplementary Fig. 8B). This release was abolished when PARP1 activity was inhibited by two chemical inhibitors, 3-aminobenzamide or Veliparib (ABT-888; Supplementary Fig. 8C).

We conclude that at senescence in NHEKs, the decrease in PARP1 expression and activity does not abolish the recruitment of XRCC1 at SSBs but impairs its phosphorylation by CK2α; the downstream recruitment of the repair enzymes is blocked and XRCC1 remains linked to the foci. Consequently, the XRCC1 foci become abnormally large and persistent, and the breaks remain unrepaired.

### Persistent XRCC1 foci engage a p38MAPK-p16-Rb pathway

**Figure 5:**
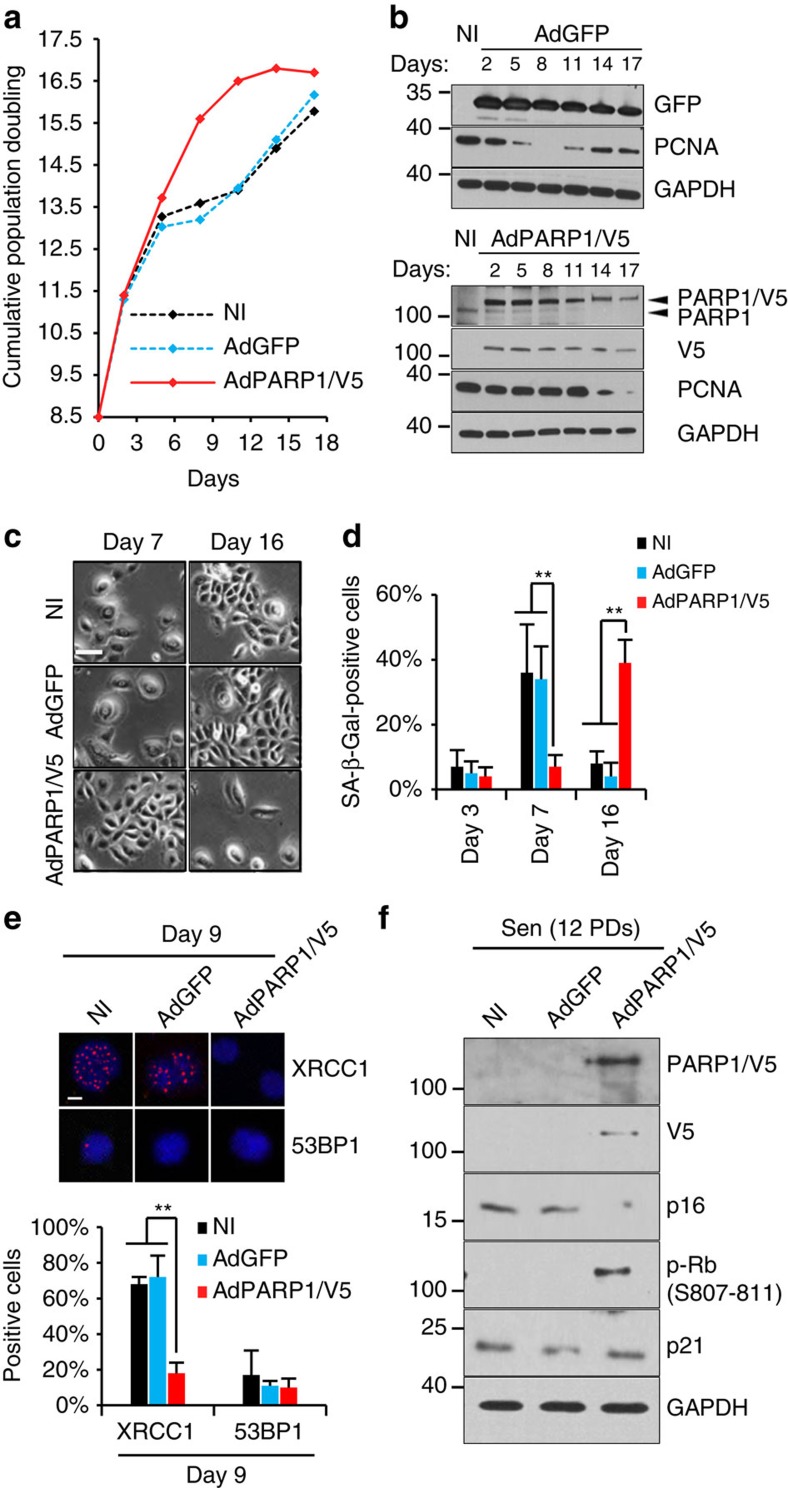
Restoring PARP1 expression resumes SSBR and delays senescence. Exponentially growing NHEKs (donor 1MC; 8.5 PDs) were infected with adenoviral vector encoding PARP1 (AdPARP1)/V5 or adenovirus encoding green fluorescence protein (AdGFP) or kept non-infected (NI). (**a**) Growth curve. (**b**) Western blot analysis of the efficacy of infection. PARP1, V5, GFP, PCNA (proliferative index) and GAPDH (loading control) levels were analysed in total cell extracts. (**c**) Representative images of cell morphology at days 7 and 16 post-infection. Scale bar=50 μm. (**d**) Percentage of SA-*β*-Gal-positive cells at days 3, 7 and 16 post-infection. The bar chart represents the mean±s.d. of five counts. (**e**) Immunofluorescence detection of XRCC1 and 53BP1. Upper panel: representative ApoTome microscopy images, scale bar, 10 μm. Lower panel: XRCC1 foci and 53BP1 foci-positive cells were automatically counted with ImageJ in 10 independent microscopic fields for a total of at least 200 cells. The bar chart represents the mean±s.d. of each 10 counts. The results are representative of two independent experiments. (**f**) Senescent NHEKs (donor 1MC) were infected as in **a**–**d**. Western blot analysis of PARP1, V5, p16, p-Rb, p21 and GAPDH (loading control) levels in total cell extracts. ExpG, exponentially growing cells; Sen, cells at the senescence plateau. The exact PDs at which cells were taken is indicated.

We then wondered whether the unrepaired SSBs could signal for the senescent cell cycle arrest. To address this question, we restored PARP1 expression in pre-senescent NHEKs. This delayed the onset of senescence by 9 days and 3 PDs (Fig. 5a–d) in correlation with a drastic decrease in XRCC1 foci but no change in 53BP1 foci (Fig. 5e). We then restored PARP1 expression in already senescent NHEKs. P16 upregulation and Rb hypophosphorylation were abrogated, whereas the expression of p21 remained unchanged (Fig. 5f). This demonstrates that the p16-dependent cell cycle arrest of senescent NHEKs occurs in consequence to the SSBR default.

**Figure 6:**
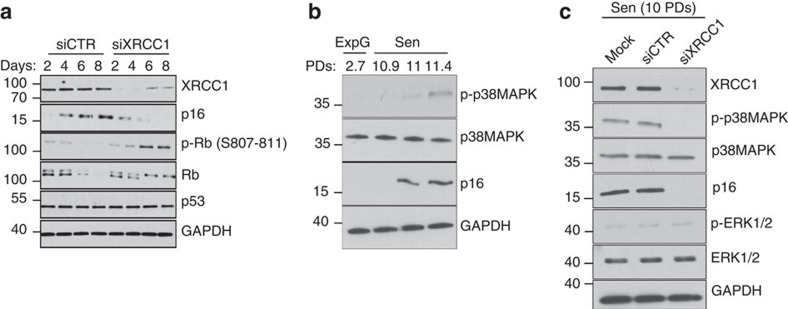
XRCC1 foci activate the p16/Rb pathway through p38MAPK. (**a**) Pre-senescent NHEKs (donor 1MC; 10 PDs) were transfected with a pool of control or anti-XRCC1 siRNAs. Western blot analysis of XRCC1, p16, Rb, phosphorylated Rb (p-Rb S807–811), p53 and GAPDH (loading control) levels in total cell extracts on days 2, 4, 6 and 8 post-transfection. (**b**) Western blot analysis of phosphorylated p38MAPK (p-p38MAPK), p38MAPK, p16 and GAPDH (loading control) in exponentially growing and senescent NHEKs (donor 1MC). (**c**) Senescent NHEKs (donor 1MC) were transfected with a pool of control or anti-XRCC1 siRNAs and analysed by western blot for XRCC1, phosphorylated ERK1/2 (p-ERK1/2), ERK1/2, phosphorylated p38MAPK (p-p38), p38MAPK, p16 and GAPDH (loading control). ExpG, exponentially growing cells; Sen, cells at the senescence plateau. The exact PDs at which cells were taken are indicated.

We then hypothesized that the enlarged and persistent XRCC1 foci could be the starting point of a signalling cascade leading to p16 upregulation. To assay this hypothesis, we avoided the formation of the XRCC1 foci by invalidating XRCC1 with siRNAs in pre-senescent NHEKs. This prevented from p16 upregulation and Rb hypophosphorylation at senescence (Fig. 6a). Since p38MAPK (MAPK14) was reported to be involved in stress-induced senescence^42^,^43^,^44^,^45^,^46^, we hypothesized that it could participate in the cascade emanating from the XRCC1 foci. We first checked that p38MAPK was activated at senescence (Fig. 6b). Then, we invalidated XRCC1 in senescent NHEKs and examined the impact on the activation of p38MAPK. It was almost completely abrogated, whereas phosphorylation of ERK1/2 was unaffected (Fig. 6c).

Altogether, these results indicate that the persistent XRCC1 foci engage a signalling pathway involving p38MAPK and leading to p16 upregulation and onset of senescence.

### Accumulation of unrepaired SSBs is sufficient to induce PSNE

**Figure 7:**
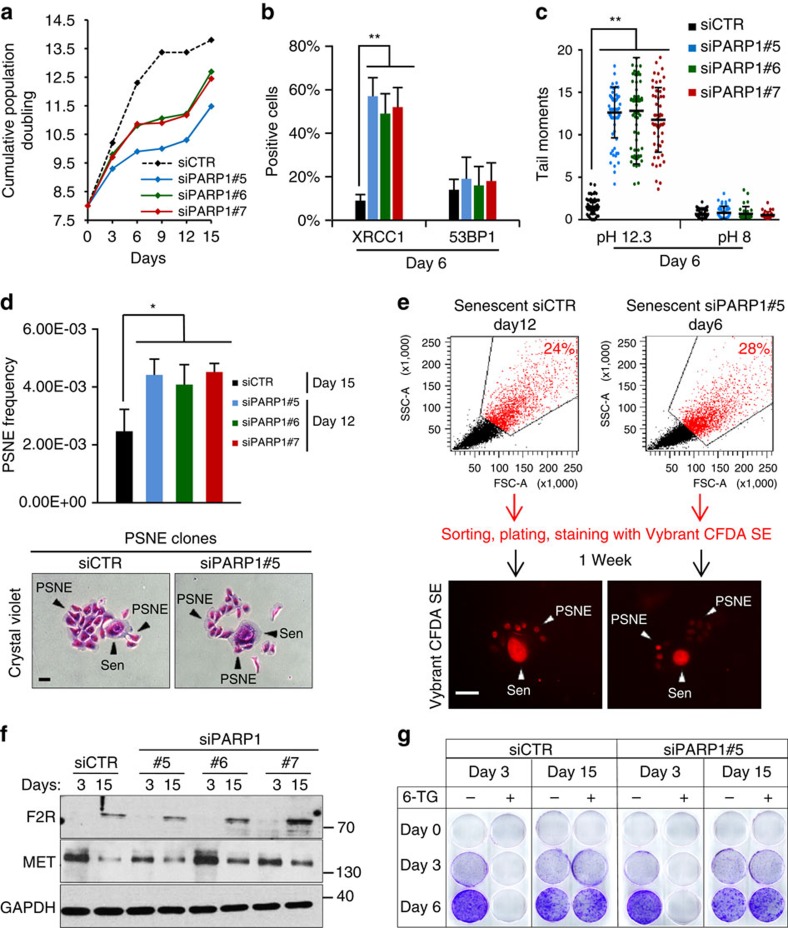
Accumulation of unrepaired SSBs is sufficient to cause PSNE. Exponentially growing NHEKs (donor 1MC; 8 PDs) were transfected with three different siRNAs directed against PARP1 or a pool of four control siRNAs. The efficiency of the siRNAs is given in Supplementary Fig. 9A. (**a**) Growth curves. (**b**) XRCC1 and 53BP1 immunofluorescences performed at day 6 post-transfection. Positive cells were automatically counted with ImageJ in five independent microscopic fields for a total of at least 100 cells for each case. The bar chart represents the mean±s.d. of each five counts. (**c**) Neutral (pH 8) and alkaline (pH 12.3) comet assays performed in tandem at day 6 post-transfection. Tail moments of 50 comet-positive cells were quantified. Scatter dot plots represent the mean±s.d. of all measures. (**d**) Upper panel: measure of PSNE frequency performed as described in Methods section at the indicated time. The given results are the mean±s.d. of counts of PSNE clones performed in five independent culture dishes. Lower panels: representative photomicrographs of PSNE clones stained with Crystal violet. Scale bar, 50 μm. (**e**) Upper panel: siCTR- and siPARP1-transfected NHEKs were analysed by FACS according to their size and granularity. About 30% of the largest and most granular cells were sorted, plated at low density, stained with the filiation tracer CFDA SE and monitored for emergence which occurred about one week later. Lower panel: representative images of PSNE clones which have inherited the fluorescent tracer from their senescent mother cell. Scale bar, 50 μm. (**f**) Western blot analysis of F2R, MET and GAPDH (loading control) levels in total cell extracts at the given time post-transfection. (**g**) HPRT assays performed at the given time post-transfection. The quantification of the results is given in Supplementary Fig. 14.

The persistence of unrepaired SSBs at senescence could induce mutations and consequently be responsible for PSNE. To test this hypothesis, we invalidated PARP1 by RNA interference in exponentially growing NHEKs and monitored them for senescence and PSNE. Premature senescence associated with p16/Rb activation occurred 6 days post-transfection, in correlation with a huge accumulation of XRCC1 foci and unrepaired SSBs (Fig. 7a–c, Supplementary Fig. 9). Importantly, this premature senescence plateau was followed by the emergence of clones of cells that reproliferated (Fig. 7a, Supplementary Fig. 9A–C). The emergence frequency, measured as the number of clones by initially plated senescent cells, was 1.6-fold higher than the control one (Fig. 7d). To verify that emergent clones were generated by fully senescent progenitors, we performed a filiation tracer assay. Senescent cells were sorted according to their large size and high granularity, plated at low density, stained with CFDA SE and monitored for PSNE. The emerging clones that appeared around senescent cells about 1 week later were positive for the filiation tracer, proving they came from the division of a stained mother senescent cell (Fig. 7e). Finally, to determine whether siPARP1-induced PSNE cells were transformed, we examined the expression of F2R and MET. They were expressed in siPARP1-induced emergent cells as in control PSNE cells (Fig. 7f). Moreover, HPRT assays indicate that the siPARP1-induced emergent cells were mutated as the control PSNE cells (Fig. 7g). To further support these results, we treated exponentially growing NHEKs with the PARP1 inhibitor 3-AB. This induced, in a dose-dependent manner, a p16-dependent premature senescence associated with SSB accumulation (Supplementary Fig. 10A–E). Despite continuing the inhibitory treatment, the premature senescence was followed by an emergence of transformed cells (Supplementary Fig. 10A,F and G). Finally, we inhibited the p38MAPK in senescent NHEKs using SB203580. This led to a decrease in p16 expression and to an increase in PSNE frequency (Supplementary Fig. 11). Taken together, these results indicate that the accumulation of unrepaired SSBs at senescence favours PSNE.

### SSBs and defective SSBR are subsequent to oxidative stress

**Figure 8:**
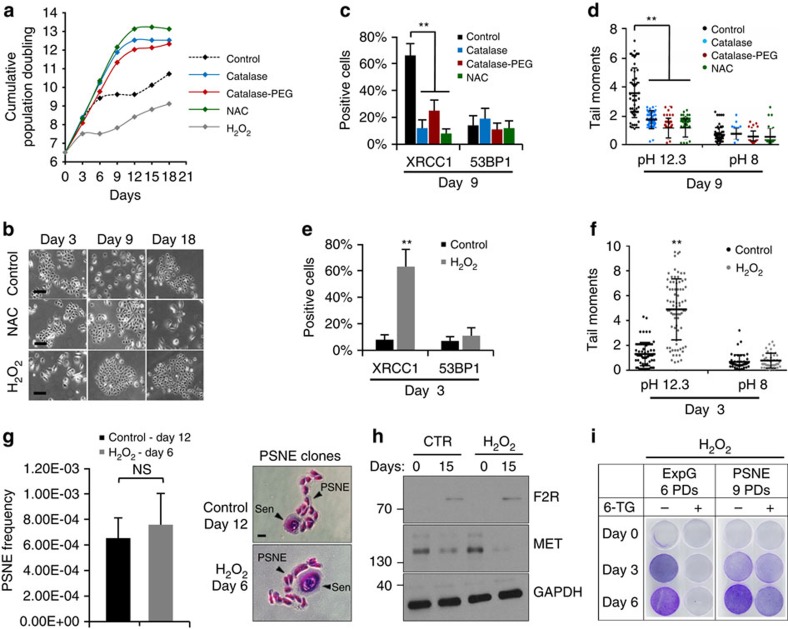
Oxidative stress resumes SSBR default-induced senescence and PSNE. Exponentially growing NHEKs (donor 1MC; 6.5PDs) were treated daily or not either with 1,000 U ml^−1^ of catalase, 1,000 U ml^−1^ of catalase-PEG, 1 mM of *N*-acetylcysteine (NAC) or 20 μM H_2_O_2_. The efficacy of the antioxidants is given in Supplementary Fig. 12B. (**a**) Growth curves. (**b**) Representative images of cell morphologies on days 3, 9 and 18 of the treatment. Scale bar, 50 μm. (**c**,**e**) Analysis of XRCC1 and 53BP1 foci-positive cells performed on antioxidant-treated and H_2_O_2_-treated cells at respectively days 9 and 3 after the beginning of the treatment. Positive cells were automatically counted with ImageJ in 10 independent microscopic fields for a total of at least 200 cells. The bar chart represents the mean±s.d. of each 10 counts. The results are representative of two independent experiments. (**d**,**f**) Neutral (pH 8) and alkaline (pH 12.3) comet assays performed in tandem on antioxidant-treated and H_2_O_2_-treated cells at respectively days 9 and 3 after the beginning of the treatment. Scatter dot plots represent the mean±s.d. of 50 measures. (**g**) Measure of PSNE frequency in control and H_2_O_2_-treated NHEKs as described in Methods section. Left: bar chart represents the mean±s.d. of counts performed in five independent culture dishes. Right: representative photomicrographs of PSNE clones stained with Crystal violet. Scale bar, 50 μm. (**h**) Western blot analysis of F2R, MET and GAPDH (loading control) levels in total cell extracts of control and H_2_O_2_-treated NHEKs at the given days post-treatment. (**i**) HPRT assays performed in H_2_O_2_-treated NHEKs at the given PDs. The quantification of the results is given in Supplementary Fig. 14.

We next wanted to determine whether the SSBs were generated by oxidative stress. First, a kinetic recording of SSB, DSB and reactive oxygen species (ROS) levels revealed that SSBs and ROS are simultaneously accumulated at just the beginning of the senescence plateau (Supplementary Fig. 12A). Second, exponentially growing NHEKs were treated with antioxidants, namely catalase, an enzyme which dismutates H_2_O_2_, catalase-PEG, a modified form of catalase designed to enter into cells, or *N*-acetylcysteine a general antioxidant (see Supplementary Fig. 12B for their efficacy). All three antioxidants delayed the occurrence of the senescence plateau by at least 9 days and 3.5 PDs (Fig. 8a,b, Supplementary Fig. 12C,D) and induced a clear decrease in SSB level without affecting DSBs (Fig. 8c,d). Importantly, no PSNE clones appeared in the antioxidant-treated cultures (Fig. 8a).

In parallel, exponentially growing NHEKs were daily treated with 20 μM H_2_O_2_. This induced premature senescence after 3 days (Fig. 8a, Supplementary Fig. 12C,D). Comet assays and XRCC1/53BP1 immunofluorescences revealed that this H_2_O_2_-induced premature senescence was accompanied by a significant increase in SSBs, whereas no DSBs were generated (Fig. 8e,f). Once the premature senescence plateau established, we stopped the H_2_O_2_ treatment and monitored the culture for PSNE. Clones appeared 7 days later (Fig. 8g, Supplementary Fig. 12C,D). They expressed transformation markers (Fig. 8h) and contained mutations (Fig. 8i).

Finally, we also wondered whether PARP1 expression could be controlled by oxidative stress as well. We therefore examined PARP1 mRNA levels after oxidant and antioxidant treatments. They were decreased after 9 h of H_2_O_2_ treatment and, conversely, increased after 9 h of *N*-acetylcysteine treatment (Supplementary Fig. 12E).

Therefore, the ROS which accumulate at senescence act both as the clastogenic chemical agents that generate the SSBs and as signalling molecules involved in the downregulation of the *parp1* gene.

### SSBR and DDR foci in aged skin

To assess the relevance of the above results to aging, we performed immunohistochemical investigations in human skin sections from three healthy young versus four aged donors (Supplementary Table 2). We first checked the accumulation of senescent cells with age by searching for p16 overexpression (for the specificity of the antibody, see Supplementary Fig. 3). We observed p16-positive cells only in the epidermis where their number increased from about 1% in young skin to 8% in aged skin (Supplementary Fig. 13).

**Figure 9:**
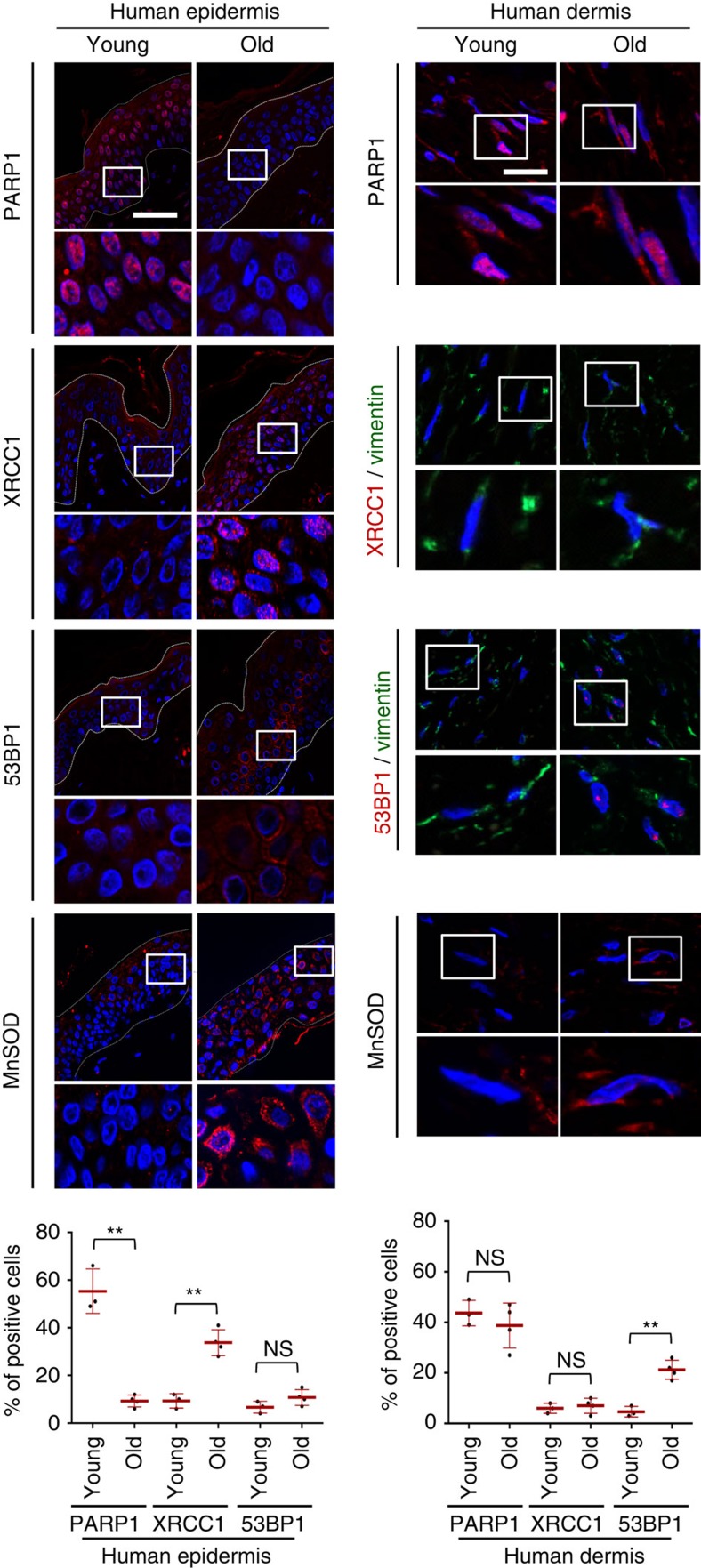
Detection of DNA damage markers in skin samples of different ages. PARP1, XRCC1/Vimentin, 53BP1/Vimentin and MnSOD immunohistofluorescences performed in sections of skin samples from healthy human young (*n*=3) and old donors (*n*=4; see Supplementary Table 2). Upper panels: representative ApoTome microscopy images for epidermis and dermis of a young (nu 32645/09, 34 years old) and an aged donor (nu 9238/09, 75 years old). Scale bar, 50 μm. The square delimits the below image at higher magnification. Lower panels: scatter dot plots indicating the percentage of positive epidermal and dermal cells in young and aged skin. Positive cells were counted in 10 independent microscopic fields for a total of at least 150 cells. The given results are the mean±s.d. of the means in the 3–4 donors.

We then performed immunohistodetections of PARP1, XRCC1, 53BP1 and Vimentin (to highlight fibroblasts within the dermal extracellular matrix). We detected nuclear PARP1 in 55% of epidermal cells of young donors, but only in 10% of epidermal cells of aged donors. In correlation, >30% of epidermal cells of aged skins displayed XRCC1 foci compared with only about 10% in young skins. Less than 10% of epidermal cells displayed 53BP1 foci, without any significant change with age. Conversely, almost all fibroblasts of the dermis were positive for PARP1 and only about 5% displayed XRCC1 foci without any significant change with age. About 20% of them in aged skins displayed one or two 53BP1 foci compared with only 5% in young skins (Fig. 9).

Subsequently, we wondered whether the epidermis could suffer from an oxidative stress increasing with aging. Since we had previously shown that, in keratinocytes, senescence is induced by NF-κB activation, MnSOD (SOD2) upregulation and H_2_O_2_ overproduction^24^,^25^, we investigated the staining pattern of MnSOD. In aged skin, almost all epidermal cells displayed an increment in MnSOD intensity. In contrast, we did not detect any apparent change with aging in dermal fibroblasts (Fig. 9).

Therefore, the skin acquires the same oxidative stress, the same decrease in PARP1 expression and the same DNA breaks during the process of aging *in vivo* as during senescence *in vitro*.

### Senescent HMECs generate PSNE cells as NHEKs

In order to determine whether the new pathways highlighted above can be generalized to epithelial cells other than NHEKs, we redrawn some of the key experiments in human mammary epithelial cells (HMECs). These cells were shown to display a growth plateau, referred as senescence, selection, M0 or stasis, followed by an emergence of cells having acquired genomic changes^22^.

**Figure 10:**
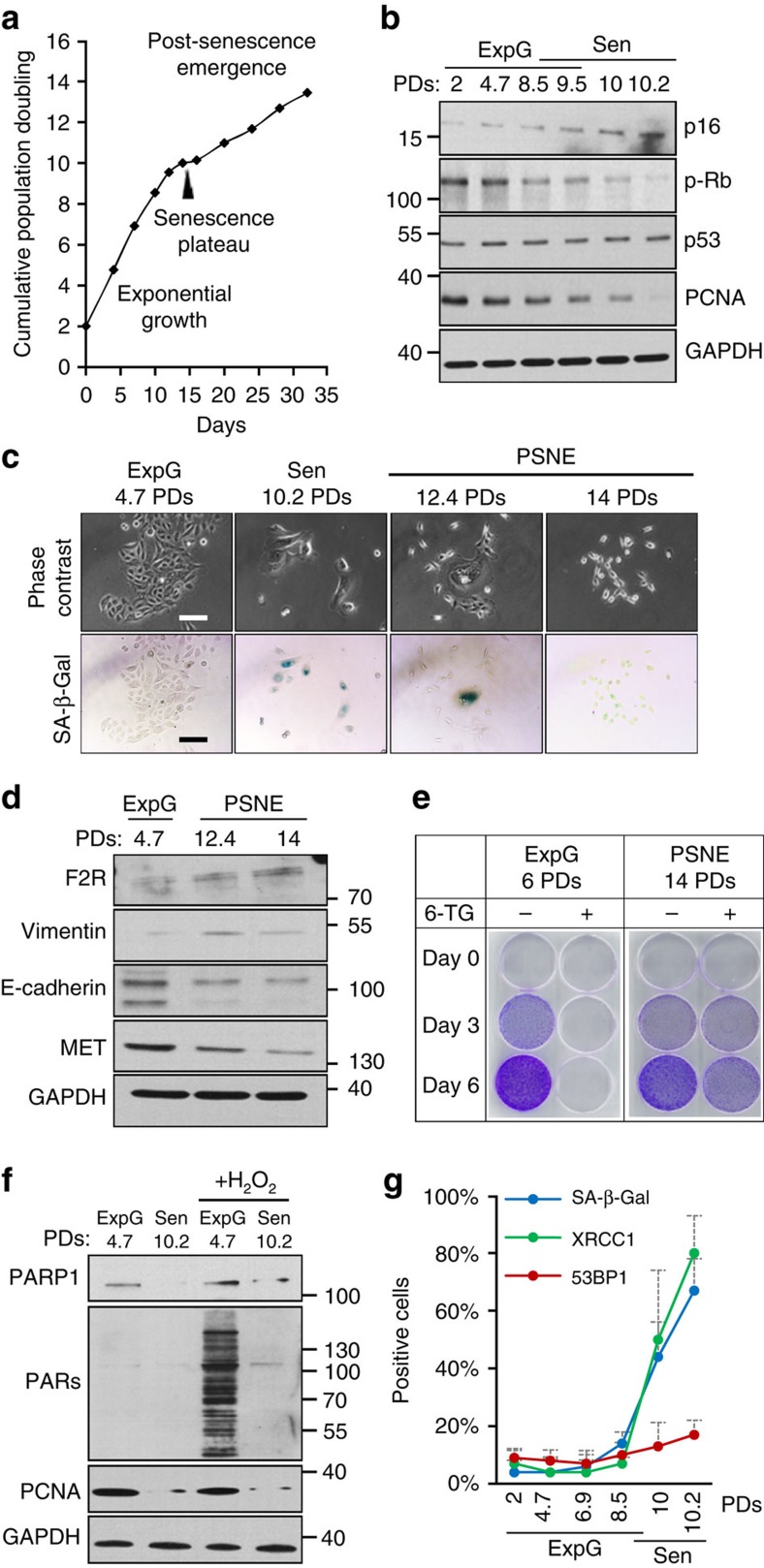
Senescence and post-senescence emergence of HMECs. (**a**) Growth curve of HMECs. (**b**) Western blot analysis of p16, phosphorylated Rb (p-Rb (S807–811)), p53, PCNA (proliferative index) and GAPDH (loading control) levels in total cell extracts. (**c**) Upper panel: morphology of HMECs observed by phase-contrast microscopy. Lower panels: images of SA-*β*-Gal-stained cells. Scale bar, 50 μm. (**d**) Western blot analysis of F2R, MET, E-cadherin, vimentin and GAPDH (loading control) levels in total cell extracts. (**e**) HPRT assays. The quantification of the results is given in Supplementary Fig. 14. (**f**) Western blot analysis of PARP1, PAR, PCNA (proliferative index) and GAPDH (loading control) levels in total extracts of exponentially growing and senescent HMECs treated or not with 100 μM H_2_O_2_ at 4 °C for 10 min and then placed at 37 °C for 5 min. (**g**) Quantification of SA-*β*-Gal, XRCC1 and 53BP1 foci-positive cells accumulation along the culture of HMECs. SA-*β*-Gal-positive cells were counted in five independent microscopic fields for a total of at least 100 cells. XRCC1 or 53BP1 foci-positive cells were automatically counted with ImageJ in 5–10 independent microscopic fields for a total of at least 100 cells at each point. Each point represents the mean±s.d. of all counts. ExpG, exponentially growing cells; Sen, cells at the senescence plateau. The exact PD at which cells were taken is indicated.

We first verified that, in our hands, HMECs were able to enter a bona fide senescence plateau and then generate post-senescence emergent cells (Fig. 10a–c). We then examined which cell cycle arrest pathway was activated at senescence. We show that, as NHEKs, HMECs at senescence upregulate p16 and hypophosphorylate Rb. P53 levels remained unchanged (Fig. 10b). We characterized the post-senescent emerging cells and show that they express the same transformation markers as NHEKs, that is, an increase in F2R and vimentin expression and a decrease in E-cadherin and MET (Fig. 10d). Moreover, using HPRT assays, we show that, as NHEKs, post-senescent HMECs are mutated (Fig. 10e).

We then examined the expression and activity of PARP1 and searched for XRCC1 foci. We found that, as NHEKs, senescent HMECs downregulate PARP1 and are compromised for PARs synthesis on a H_2_O_2_ challenge (Fig. 10f). In consequence, they massively accumulate XRCC1 foci (Fig. 10g). As NHEKs, they do not significantly accumulate DDR foci (Fig. 10g).

## Discussion

Senescence is known as a state of irreversible cell cycle arrest resulting from the accumulation of persistent telomeric and non-telomeric DDR foci which activate the p53/p21 tumor-suppressor pathway^47^. In this report, we confirm these results for fibroblasts but present new *in vitro* and *in vivo* data concerning keratinocytes and mammary epithelial cells. We show that epithelial cells at senescence do not suffer from significantly shortened telomeres, do not accumulate DSBs, nor do they activate the DDR pathway and therefore do not significantly activate the p53/p21 pathway. Instead, they suffer from a decrease in PARP1 expression, which compromises the repair of SSBs generated by oxidative stress. This induces the persistence of SSBR foci which engage a signalization cascade leading to p38MAPK activation and to p16 upregulation. These results qualify the paradigm on the role of DNA damage in senescence: they confirm the persistence of unrepaired DNA damage and the formation of persistent DNA damage foci as a universal cause of senescent cell cycle arrest; however, the nature of the damage, the nature of the persistent foci and the cell cycle arrest pathway would be cell-type specific.

It is known for several years that the p16/Rb pathway has a central role in inducing senescence in epithelial cells^48^,^49^,^50^. Some studies had suggested that oxidative stress is one trigger for the activation of the p16/Rb pathway^47^,^51^, but the precise mechanisms were not determined. Here we demonstrate the causal links between the accumulation of ROS, SSBs, the activation of the p16/Rb pathway and the cell cycle arrest. Moreover, we show that ROS work at least in two synergistic ways at senescence. First, they are the chemical agents that broke the sugar-phosphate backbone of DNA and generate the SSBs. Second, they also cause the downregulation of PARP1 that makes the SSBR pathway defective. Few data on PARP1 expression regulation are presently available. Studies of the *parp1* promoter have revealed that it is TATA-box less, but rich in CpG islets with binding sites for SP1 (ref. 52), YY1 (ref. 53) and ETS (ref. 54). The downregulation of PARP1 on oxidative stress is counter-intuitive because it increases the harmful effects of ROS. However, besides its role in SSBR, PAR synthesis fulfils several other functions including caspase-independent cell death such as necroptosis or parthanatos^55^,^56^. Therefore, the downregulation of PARP1 on sustained oxidative stress could serve to protect (senescent) cells from cell death.

The dynamics of recruitment-dissociation of SSBR proteins in senescent epithelial cells seem to freeze at the stage of XRCC1 recruitment. This is under the dependence of the PARP1 expression which is, at senescence, decreased but not completely lost. The residual PARP1 activity could be sufficient to synthesize some PARs, short, but long enough to recruit XRCC1. This is supported by a recent study showing that PARs made of only seven ADP-ribose units have a high affinity for XRCC1 (ref. 57). In contrast, the few PARs could be insufficient to recruit CK2α and phosphorylate XRCC1, a key step for both the recruitment of PNKP and APTX and for the dissociation of XRCC1 from the foci. In consequence, the breaks remain unrepaired, XRCC1 remains linked to the foci and additional XRCC1 proteins continue recruiting, making XRCC1 foci large platforms nucleating a signalization cascade involving p38MAPK.

Interestingly, the sole decrease of PARP1 expression using siRNAs in proliferating keratinocytes is sufficient to induce a premature senescence plateau, including not only the activation of the p16/Rb pathway but also all the other major characteristics of senescence, that is, cell enlargement and SA-*β*-Gal activity. This suggests that not only the cell cycle arrest, but some of the other changes characteristic of senescence may be the consequence of this XRCC1/p38MAPK signalization. Therefore, the decrease in PARP1 expression and the increase in oxidative stress are the key initiating events of senescence in epithelial cells. Persistent XRCC1 foci function as sensors of unrepaired oxidative SSBs, and p38MAPK could be a mediator protein common to several downstream effector pathways.

Remarkably, both the DDR and SSBR pathways that are activated at senescence *in vitro* in fibroblasts and keratinocytes are also activated with aging *in vivo*, and are also specific for conjunctive and epithelial tissues, respectively, at least in the skin. Moreover, oxidative stress is also preferentially important in the epidermis. The number of affected cells in skins from old donors is especially high: almost all epidermal cells are MnSOD positive and PARP1 negative, and about 40% display XRCC1 foci, whereas only 8.5% are p16 positive. This raises the question of what is the best marker of senescence for *in vivo* studies. Data regarding biomarkers of senescence in the skin are very few. In his seminal publication, Dimri reported the presence of SA-*β*-Gal-positive cells in aged human skin, but no precise quantification was done^2^. Ressler *et al*.^58^ also reported accumulation of p16-positive cells in the epidermis, but the quantification was not done in terms of percentage. A study of Wang *et al*.^4^ quantified the percentage of γH2AX foci in the mouse epidermis to 3–5%, without any change with age, in accordance with our results. The group of Sedivy reported 15–25% of cells positive for 53BP1 foci in the dermis of baboons^3^, in accordance with our results. Therefore, we propose to use XRCC1 foci in addition to p16 as a marker of epithelial cell senescence *in vivo* and *in vitro*, and we propose to restrict the use of 53BP1 or γH_2_AX foci for fibroblasts and conjunctive tissues.

Senescence is recognized as an intrinsic tumor-suppressor mechanism. This assumption relies on the stability of the cell cycle arrest, which is itself the consequence of the persistence of the DDR foci^9^. However, with respect to epithelial cells, our past and present results suggest that senescence is intrinsically both tumor suppressor and tumor promoter. These two properties, although opposite, rely on the same characteristic of the epithelial cell senescence: its transience. Indeed, in one side, for most cells, senescence ends up in autophagic cell death^7^,^26^ which is, as a tumor-suppressor mechanism, more efficient than cell cycle arrest. In another side, for a small subpopulation, senescence is followed by a re-entry in cell cycle which generates mutated, transformed and tumorigenic cells^24^. The accumulation of unrepaired SSBs is sufficient for the occurrence of this phenomenon. How unrepaired SSBs contribute to neoplastic emergence has to be investigated. One can speculate on the initiation of an inaccurate repair pathway. Since decrease in PARP1 expression is at the origin of both senescence and neoplastic emergence, PARP1 could be viewed as both a tumor promoter and a tumor-suppressor gene. In support, it was shown that a PARP1 pharmacological inhibitor was able to induce senescence in cancer cells^59^,^60^,^61^,^62^. In contrast, PARP1 deficient mice were shown to develop signs of accelerated aging with epidermal hyperplasia, carcinomas and are prone to develop tumours on exposition to base damaging agents^63^,^64^,^65^.

In conclusion, senescence results from the persistence of a DNA damage signalization, but the exact nature of the unrepaired damage could vary in different cell types depending on their repair capacities and could dictate completely different outcomes. Namely, persistent DSBs, including telomeric ones, dictate a permanent tumor-suppressor cell cycle arrest, whereas persistent SSBs are permissive to mutations and senescence evasion.

## Methods

### Cell culture and calculation of PDs

NHDFs and NHEKs were purchased from Promocell, Tebu—bio, GIBCO or Cambrex. HMECs were purchased from Bio-Whittaker. For details, see Supplementary Table 1. Cells were grown at 37 °C in an atmosphere of 5% CO_2_ and at the atmospheric O_2_ tension. NHEKs were cultured in the KGM-Gold^TM^ bulletkit medium (Clonetics). It consists of modified MCBD153 with 0.15 mM calcium, supplemented with bovine pituitary extract, epidermal growth factor, insulin, hydrocortisone, transferrin and epinephrine. Such a serum-free low-calcium medium has been shown to minimize keratinocyte terminal differentiation^66^. NHDFs were cultured in FGMTM-2 bulletkit medium. HMECs were cultured in MEGM^TM^ bullekit medium.

Cells were seeded as recommended by the supplier and subcultured at 70% confluence. The number of PDs was calculated at each passage by using the following equation: PD=log (number of collected cells/number of plated cells)/log2.

### SA-*β*-Gal assays

Cells were fixed using 2% formaldehyde/0.2% glutaraldehyde in phosphate-buffered saline for 4 min and incubated with X-Gal-containing reaction mixture as described by Dimri *et al*.^2^: 1 mg ml^−1^ X-Gal; 40 mM phosphate buffer (pH 6); 5 mM potassium ferrocyanide; 5 mM potassium ferricyanide; 150 mM NaCl; 2 mM MgCl_2_. Incubation time was 7–8 h for NHDFs and 24 h for NHEKs and HMECs. SA-*β*-Gal-positive cells were counted in 5–10 independent microscopic fields for a total of at least 100 cells for each case in all experiments.

### Reagents

Catalase (C1345), catalase-PEG (C4963) and *N*-acetylcysteine (A7250) were purchased from Sigma and diluted in phosphate-buffered saline (PBS). The used PARP inhibitors were 3-aminobenzamide (A0788, Sigma-Aldrich) and ABT-888 (Veliparib; A3002, ApexBio).

The used P38MAPK inhibitor was SB203580 (S8307, Sigma-Aldrich).

### Calculation of PSNE frequency

The PSNE frequency was calculated as follows: senescent NHEKs were plated at low density (350 cells per cm^2^) and monitored for PSNE clone appearance by carefully scanning each culture dish under a phase-contrast microscope at least twice and at different days after plating. The frequency of PSNE was calculated as the ratio of the number of recorded clones to the number of initially seeded senescent cells.

Vybrant CFDA SE Cell Tracer Kit was purchased from Life technologies (V12883, Molecular Probes).

### Filiation tracer assays

Filiation tracer assays were performed as recommended by the supplier. In brief, sorted senescent cells were incubated with 10 μM of CFDA SE probe for 30 min at 37 °C. After that, cells were fixed for 15 min at room temperature (RT) using 3.7% formaldehyde, washed with PBS and mounted in Glycergel (Dako).

### Western blotting

Cells were lysed in SDS–polyacrylamide gel electrophoresis sample buffer (50 mM Tris pH 6, 8; 4% SDS; 20% glycerol; 5% *β*-mercaptoethanol and bromophenol blue). Proteins were resolved by SDS–polyacrylamide gel electrophoresis and transferred to nitrocellulose membranes (Hybond-C Extra, Amersham). Membranes were incubated with the primary antibody. The used antibodies are listed in Supplementary Table 3 with indication of their supplier and used dilution. Secondary antibodies were peroxidase-conjugated anti-mouse IgG, anti-rabbit IgG or anti-goat IgG (715-035-151, 711-035-152 and 705-035-003 Jackson-Immuno Research Laboratories). Peroxidase activity was revealed using an ECL kit (RPN2106, Amersham Biosciences) or ECL Prime kit (RPN2236, Amersham Biosciences) or SuperSignal West Dura Extended Duration Substrate (34076, Thermo Scientific). Uncropped scans of Western blots are presented in Supplementary Figs 15–18.

### Immunofluorescence on cells and tissue sections

Cells were fixed in cold methanol/acetone (vol/vol) or PFA 4% in PBS for 10 min, and washed in PBS. Non-specific binding was blocked by incubation in 5% non-fat milk in PBS. Human skin samples were obtained from the Bonn University (Germany) anatomopathology department, according to the german regulations. They were fixed in formalin, paraffin embedded and sectioned at 10 μm. Sections were dewaxed and rehydrated according to standard procedures. Non-specific binding was blocked by incubation in 5% bovine serum albumin in PBS. Primary antibody was incubated for 1 h at 37 °C or overnight at 4 °C. The used antibodies are listed in Supplementary Table 3 with indication of their supplier and used dilution. After washings in PBS, cells or sections were incubated with Rhodamine anti-IgG Mouse (715-296-150, Jackson Immuno Research Laboratories), AlexaFluor 488 anti-IgG Rabbit (A21206; Molecular Probes), AlexaFluor 488 anti-IgG Mouse (A-21202, Molecular Probes) or AlexaFluor 555 anti-IgG Sheep (A-21436; Molecular Probes) for 60 min at RT. For double immunofluorescence, the two primary and two secondary antibodies were co-incubated. Finally, cells or sections were washed in PBS, nuclei were stained for 5 min with Hoechst (33258, Sigma-Aldrich) at 1 μg ml^−1^ and mounted in Glycergel (Dako). Optical sectioning images were taken using LSM 780 Confocal Microscope or AxioImager Z1-Apotome (Zeiss, Germany). AxioVision or ZEN softwares were used for microscope image analysis (Zeiss).

### BrdU-incorporation assays

For staining cells in S-phase, BrdU (10 280 879 001, Roche) was added to cell cultures at 20 μM for 1 h. For staining SSB foci undergoing repair, BrdU was added overnight. Cells were then fixed with 4% paraformaldehyde in PBS, permeabilized with 0.2% Triton X-100 and incubated with 40 U ml^−1^ DNase I (M6101, Promega) and 20 U ml^−1^ Exonuclease III (M1811, Promega) for 30 min at 37 °C. BrdU was revealed with anti-BrdU mouse IgG (M 0744, Dako) and Rhodamine Red-conjugated anti-mouse IgG (715-296-150, Jackson Immunoresearch laboratories).

### HPRT assays

Cells were plated at low density (700–1,500 cells per cm^2^). They were exposed to 100 μM of 6-thioguanine (6-TG; A4882, sigma) twice per day. Cells were fixed in PFA 4% in PBS on days 0, 3 and 6 post-treatment and then stained with 0.05% crystal violet in distilled water. For quantifying the results, the crystal violet was re-dissolved in 2% SDS in distilled water. The colour intensity was then quantified by measuring the absorbance at 570 nm. The quantification of the results is given in Supplementary Fig. 14.

### RNA interference

For PARP1 knockdown, three different siRNAs from the on-target plus set of four siRNA (J-006656-00-0005, Dharmacon) were used. The target sequences were: 5′- GAUUUCAUCUGGUGUGAUA -3′; 5′- GAAAACAGGUAUUGGAUAU -3′; 5′- GUUCUUAGCGCACAUCUUG -3′. For XRCC1 knockdown, a pool of four different siRNAs (L-009394-00, Dharmacon) was used. The target sequences were: 5′- AAACUCAUCCGAUACGUCA -3′; 5′- CCGCAAGCCUGAAGUAUGU -3′; 5′- GGAAUGAUGGCUCAGCUUU -3′ and 5′- AGGCAGACACUUACCGAAA -3′. For p16 knockdown, a pool of four different siRNAs (L-009394-00, Dharmacon) was used. The target sequences were: 5′- GAUCAUCAGUCACCGAAGG -3′; 5′- AAACACCGCUUCUGCCUUU -3′; 5′- UAACGUAGAUAUAUGCCUU -3′ and 5′- CAGAACCAAAGCUCAAAUA -3′. For CK2α knockdown, a pool of four different siRNAs (L-003475-00-0005, Dharmacon) was used. The target sequences were: 5′- GCAUUUAGGUGGAGACUUC -3′, 5′- GGAAGUGUGUCUUAGUUAC -3′, 5′- GCUGGUCGCUUACAUCACU -3′ and 5′- AACAUUGUCUGUACAAGGUU -3′. For PNKP knockdown, a pool of four different siRNAs (E-006783-00-0005, Dharmacon) was used. The target sequences were: 5′- CUCUGGUGUCCCAAGAUGA -3′, 5′- CCUUUGAUCCGAGGACUGU -3′, 5′- UCACCAACCAGAUGAGCAU -3′ and 5′- GGACACACUGUAUUUGGUC -3′.

In all cases, a non-targeting siRNA pool (siGENOME RISC-Free Control siRNA, Dharmacon) was used as control. Cells were transfected with 25 nM siRNAs using the Lipofectamine RNAiMAX transfection reagent (Invitrogen) in optiMEM (Gibco). After 6 h of incubation at 37 °C, the transfection medium was replaced with fresh culture medium.

### Adenoviral infection

Adenovirus encoding V5-tagged PARP1 (SL101030) and non-tagged PARP1 (SL100927) were purchased from SignaGen Laboratories. The recombinant adenovirus encoding green fluorescent protein was a gift from D. Leprince^24^. Adenovirus encoding green fluorescence protein viral titre was determined by a plaque assay on 293 cells and defined as plaque-forming units per ml. Cells were infected by adding virus stocks directly to the culture medium at an input multiplicity of 400 viral particles per cell. After 4 h of incubation at 37 °C, the infection medium was replaced with fresh culture medium.

### Cell cycle analysis by flow cytometry

BrdU (10 280 879 001, Roche) was added to cell cultures at 20 μM for 1 h. Cells were fixed with 90% ethanol overnight at 4 °C, rinsed in PBS and incubated with 2 N HCl/0,5% Triton X-100 at RT for 30 min. After that, cells were suspended in 0.1 M sodium tetraborate for 2 min. Cells were incubated with anti-BrdU mouse IgG (M 0744, Dako) for 1 h at 37 °C, washed with PBS and incubated with AlexaFluor 488 anti-IgG Mouse (A-21202, Molecular Probes) for 1 h at RT. Cells were finally incubated with PBS containing 10 μg ml^−1^ RNase A and 20 μg ml^−1^ propidium iodide for 30 min at 37 °C and then analysed by flow cytometry on BD-FACSCanto II. The results were analysed with the FACSDiva 7.0 software.

### Quantitative FISH (Q-FISH)

Q-FISH experiments were performed either on metaphases or interphasic nuclei. Metaphase spreads were obtained using a standard method. In brief, cells were incubated 1 h in Karyomax Colcemid (Invitrogen Corporation), trypsinised and incubated in a 60-mM KCl hypotonic buffer. Cells were fixed with freshly made methanol/acetic acid solution (3:1 v/v), spread onto frozen slides and air dried overnight. Then slides were post-fixed in 4% formaldehyde in PBS for 2 min, washed three times in PBS and treated with pepsin (P-7000, Sigma) at 1 mg ml^−1^ for 10 min at 37 °C at pH 2.0. After a brief wash in PBS, formaldehyde fixation and washes were repeated and the slides were dehydrated with ethanol and air dried. The hybridization mixture containing 70% formamide, the nucleic acid probes labelled with Cy3 at 0.3 μg μl^−1^ (Perceptive Biosystems, Ramsey, MN), 1% (W/V) blocking reagent (Boehringer-Mannheim, Gmbh) in 10 mM Tris pH 7.2 was laid down, a coverslip was added and DNA was denatured for 3 min at 80 °C. After 2 h hybridization at RT, slides were washed with 70% formamide/10 mM Tris pH 7.2 (2 × 15 min) and with 0.05 M Tris 0.15 M NaCl pH 7.5 containing 0.05% Tween-20 (3 × 5 min). Slides were then counterstained with 1 μg ml^−1^ 4′,6-diamidino-2-phenylindole (DAPI) and mounted in antifading solution (VectaShield, Vector Laboratories Inc., Burlingame, CA).

Cells grown on sterile coverslips were fixed with 4% formaldehyde, washed three times in PBS and incubated with RNase solution (100 μg ml^−1^) for 1 h at 37 °C. After that, they were washed three times in SSC 2% and dehydrated by 75, 95 and 100% ethanol bathes and finally air dried for 5 min. Coverslips were incubated with 200 nM TelG-Cy3 probe (F1006, Panagene Inc.) in hybridization buffer (60% formamide, 20 mM Tris-HCl, 20 mM Na2HPO4, 2% SSC and 0.1 μg ml^−1^ of salmon sperm DNA) at 80 °C for 5 min, followed by 2 h in dark at RT. Cells were then washed three times for 10 min with washing buffer (formamide 60%, SSC 2% and Tris-HCl 20 mM) and three times for 5 min with (SSC 2% and Tween 0.05%). Finally, nuclei were stained for 5 min with Hoechst (33258, Sigma-Aldrich) at 1 μg ml^−1^, and mounted in Glycergel (Dako). Optical sectioning images were taken with an Axioplan2 (Germany) microscope equipped with an Apotome device. Telo-PNA fluorescence of >50 nuclei for each condition were analysed using the TFL-TELO programme.

### Comet assays

For each condition, 2,000 cells were suspended in 80 μl of 0.5% low-melting point agarose at 42 °C. The suspension was immediately laid onto a comet slide (Trevigen Inc.). Agarose was allowed to solidify at 4 °C for 20 min. The comet slides were then immersed in prechilled lysis solution (1.2 M NaCl, 100 Mm EDTA, 10 mM Tris, 1% Triton (pH 10)) at 4 °C, for 90 min in the dark. After this treatment, comet slides were placed in a horizontal electrophoresis unit and allowed to equilibrate in electrophoresis buffer for 10 min at 4 °C, in the dark. For assessing both SSBs and DSBs, the migration was performed in 300 mM NaOH, 1 mM EDTA (pH=12.3). After migration, the slides were neutralized with 0.4 M Tris (pH=7.5). To detect DSBs, the electrophoresis buffer was 89 mM Tris, 89 mM boric acid and 2 mM EDTA (pH 8). The migration was performed at 30 V for 20 min and 40 V for 25 min for pH=12.3 and 8, respectively. After migration, the slides were stained with SYBR Green (X1000; Molecular Probes) according to manufacturer's recommendations. Tail moments (=tail length × DNA in the tail/total DNA) were analysed using the Tritek Comet Score freeware.

### Measure of ROS concentration

Steady-state ROS concentration was measured using non-fluorescent H_2_DCFDA (2′,7′-dichlorofluorescein diacetate; D399, Molecular Probes), which diffuses across membranes and is oxidized to fluorescent DCF. Cells were rinsed in PBS, incubated with H_2_DCFDA diluted in PBS at 5 μM for 15 min at 37 °C. After that, fluorescence was measured using a fluorometer (Fluostar Optima from BMG Labtech) with FITC filters.

### Reverse-transcription quantitative real-time PCR

RNAs were isolated with the Nucleospin kit (Macherey-Nagel). Reverse transcription was carried out for 1 h at 55 °C with 1 μg total RNA, oligodT primers, dNTPs and Superscript II reverse transcriptase (Invitrogen; 200 units). Primers for PCR were designed with the qPrimerDepot software (http://primerdepot.nci.nih.gov/). Primer sequences were: *parp1* forward 5′- GCCCTAAAGGCTCAGAACGA -3′ and reverse 5′- CAGAAGGCACTTGCTGCTTG -3′; *twist* forward 5′- GGCTCAGCTACGCCTTCTC -3′ and reverse 5′- TCCATTTTCTCCTTCTCTGGAA -3′, *slug* forward 5′- TCGGACCCACACATTACCTT -3′ and reverse 5′- TGACCTGTCTGCAAATGCTC -3′, *EAR* forward 5′- GAGGCTGAGGCAGGAGAATCG -3′ and reverse 5′- GTCGCCCAGGCTGGAGTG -3′.

The PCR protocol was as recommended for the Mx3005P Real-time PCR System (Stratagene). Accumulation of PCR products was measured by SYBR green fluorescence (SYBR Green master mix; Applied Biosystems). Raw data analysis was performed with the MxPro software (Agilent). All target gene transcripts were normalized to the *EAR* transcripts. All experiments were conducted at least three times with independent RNA isolations.

### Statistical analyses

Statistical analyses were done using Student *t*-test. Significant differences are indicated with * when *P*<0.5 and with ** when *P*<0.01. When *P*>0.5, differences are considered as non-significant and are indicated as NS.

## Additional information

**How to cite this article:** Nassour, J. *et al*. Defective DNA single-strand break repair is responsible for senescence and neoplastic escape of epithelial cells. *Nat. Commun.* 7:10399 doi: 10.1038/ncomms10399 (2016).

## Supplementary Material

Supplementary Information Supplementary Figures 1-18 and Supplementary Tables 1-3
