# Supplementary material for: Defective DNA single-strand break repair is responsible for senescence and neoplastic escape of epithelial cells
Source: Nat Commun. 2016 Jan 29;7:10399. doi: 10.1038/ncomms10399 (PMC4740115; doi:10.1038/ncomms10399)
Supplement: Supplementary Information — Supplementary Figures 1-18 and Supplementary Tables 1-3 [file ncomms10399-s1.pdf]

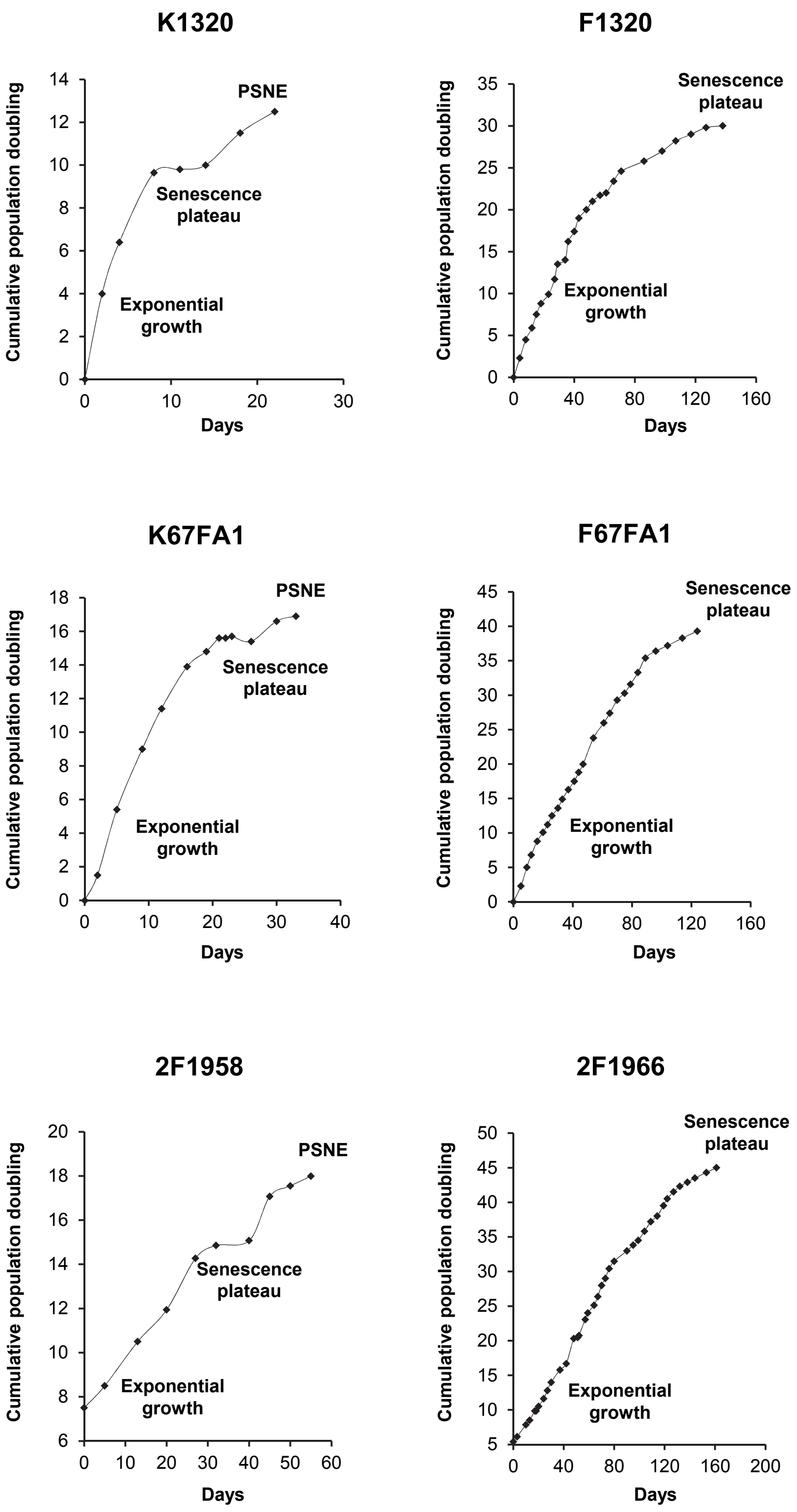

Supplementary Figure 1: Growth curves of the three NHDFs-NHEKs couples used in this study. See supplemental table 1 for the characteristics of each donor.

A

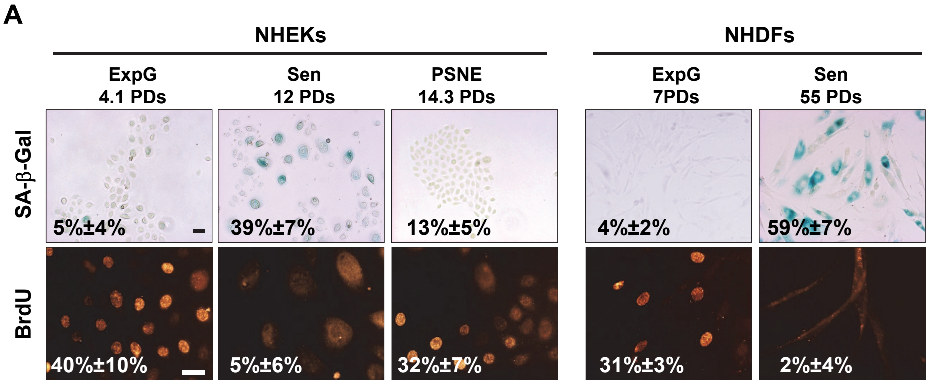

T- test	NHEKs ExpG/Sen	NHEKs Sen/PSNE	NHDFs ExpG/Sen
SA-β-Gal	P= 1.91933E-05	P= 9.73507E-05	P= 2.15564E-05
BrdU staining	P= 0.000122062	P= 3.88543E-05	P= 3.7863E-07

B

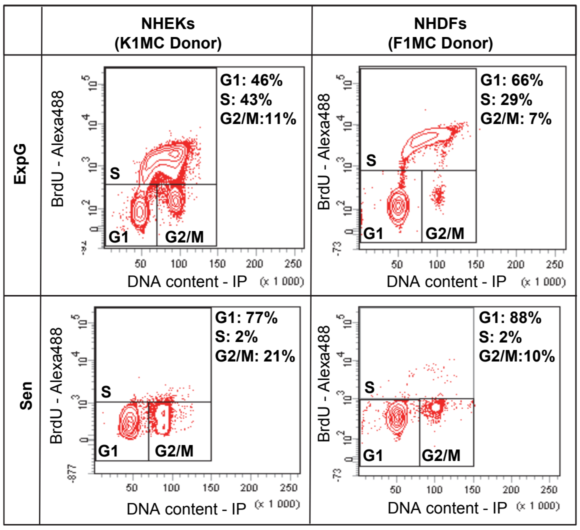

Supplementary Figure 2: Characteristics of senescent NHEKs and NHDFs (complements to Figure 1).

(A) Upper panels: images of SA-β-Gal-stained NHEKs and NHDFs (donor 1MC). Scale bar=50µm. The means ± SD of SA-β-Gal-positive cells are indicated as inserts. Lower panels: BrdU incorporation assays with quantification of BrdU-positive cells. Scale bar=10µm. Positive cells were counted in 5 independent microscopic fields for a total of at least 100 cells for each case. The results given as inserts are the mean +/- SD of all counts. The statistical analyses are given in the table below. (B) Dot plots of cell cycle distribution of exponentially growing and senescent NHEKs and NHDFs (donor 1MC) analyzed by flow cytometry (F1MC (ExpG: 11 PDs – Sen: 58 PDs), K1MC (ExpG: 3 PDs – Sen: 12.4 PDs)). Percentages of cells in G0/G1, S, and G2/M phases are indicated. The experiment was performed with 3 couples of NHDFs-NHEKs from 3 different donors. The statistical analysis of the 3 experiments is given in Fig.1B.

ExpG=exponentially growing cells; Sen=cells at the senescence plateau. The exact PDs at which cells were taken is indicated.

A

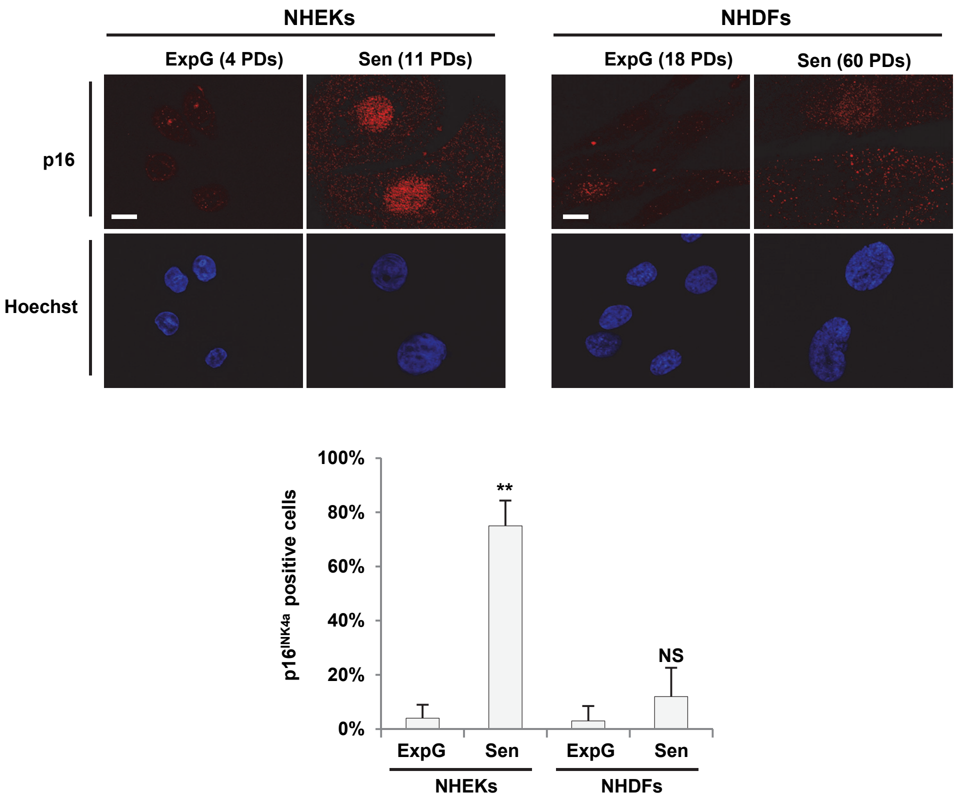

B

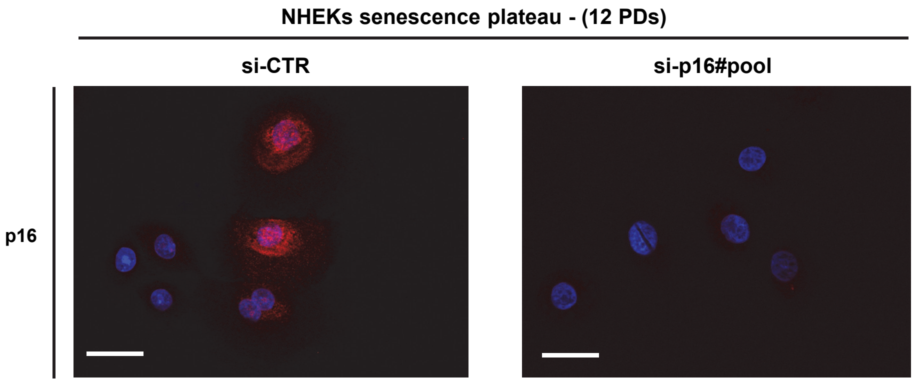

Supplementary Figure 3: NHEKs but not NHDFs upregulate p16 at senescence (complements to Figure 1).

(A) Immunofluorescence detection of p16 in exponentially growing and senescent NHEKs and NHDFs (donor 1MC). Upper panels: Representative ApoTome microscopy images. Scale bar=10µm. Lower panel: Quantification of p16-positive cells. Positive cells were automatically counted with ImageJ in 5 independent microscopic fields for a total of at least 50 cells. The bar chart represents the mean ± SD of each 10 counts. (B) Checking for the specificity of the p16 antibody. Senescent NHEKs (donor 1MC) were transfected by a pool of control or p16 siRNAs and processed for immunofluorescence with the anti-p16 antibody (550834, BD Pharmingen). Representative ApoTome microscopy images, scale bar=20µm.

ExpG=exponentially growing cells; Sen=cells at the senescence plateau. The exact PDs at which cells were taken is indicated.

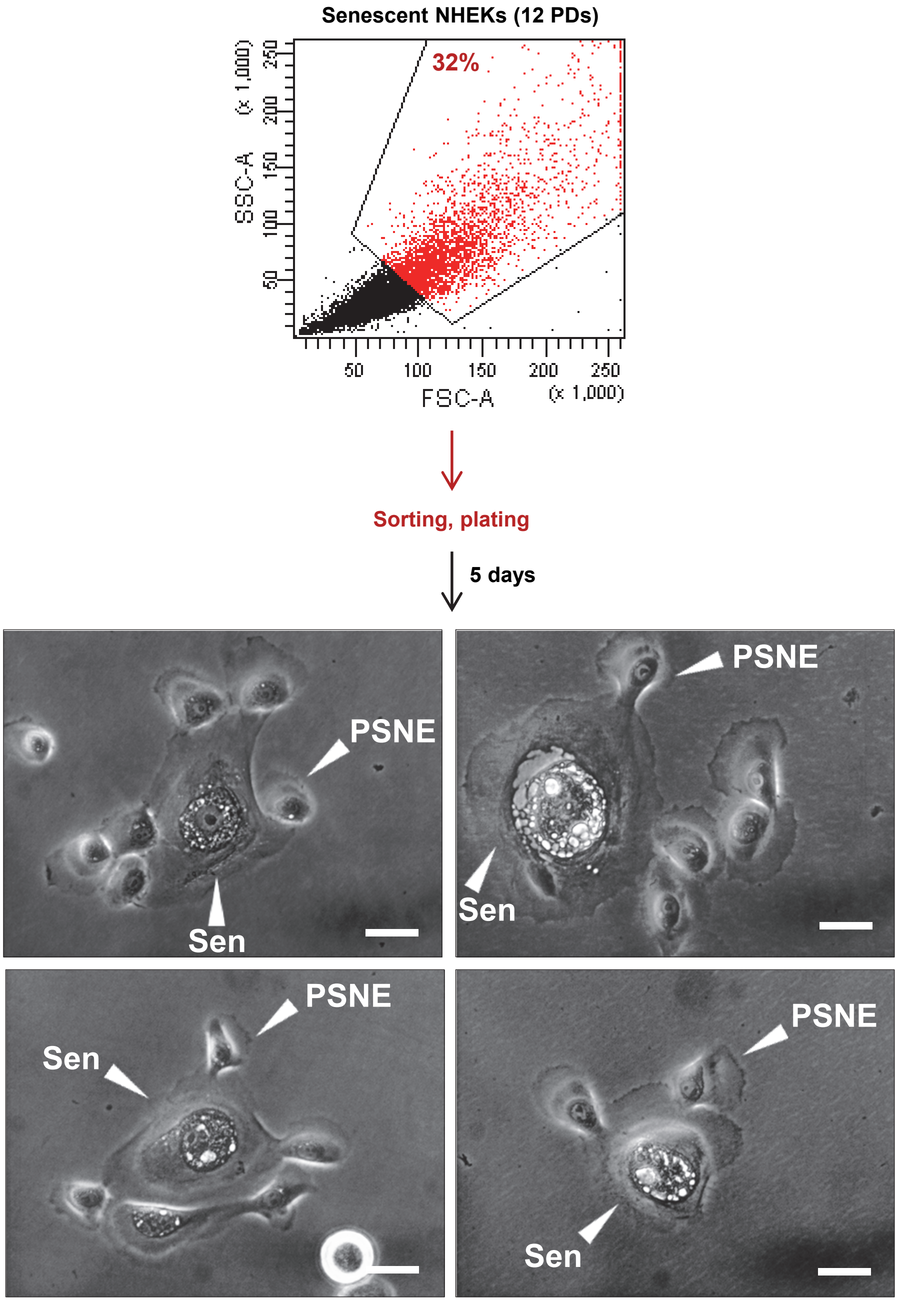

Supplementary Figure 4: PSNE cells are generated from fully senescent cells (complements to Figure 1).
Senescent NHEKs (donor 1MC) at 12 PDs were analyzed by flow cytometry according to their size (FSC-A) and granularity (SSC-A). Thirty-two percent of the largest and most granular cells were sorted, plated at low density and monitored for PSNE which occurred 5 days later. Representative images by phase contrast microscopy of PSNE clones clearly showing the remaining links between PSNE cells and their senescent mother cell. Scale bar= 50µm.

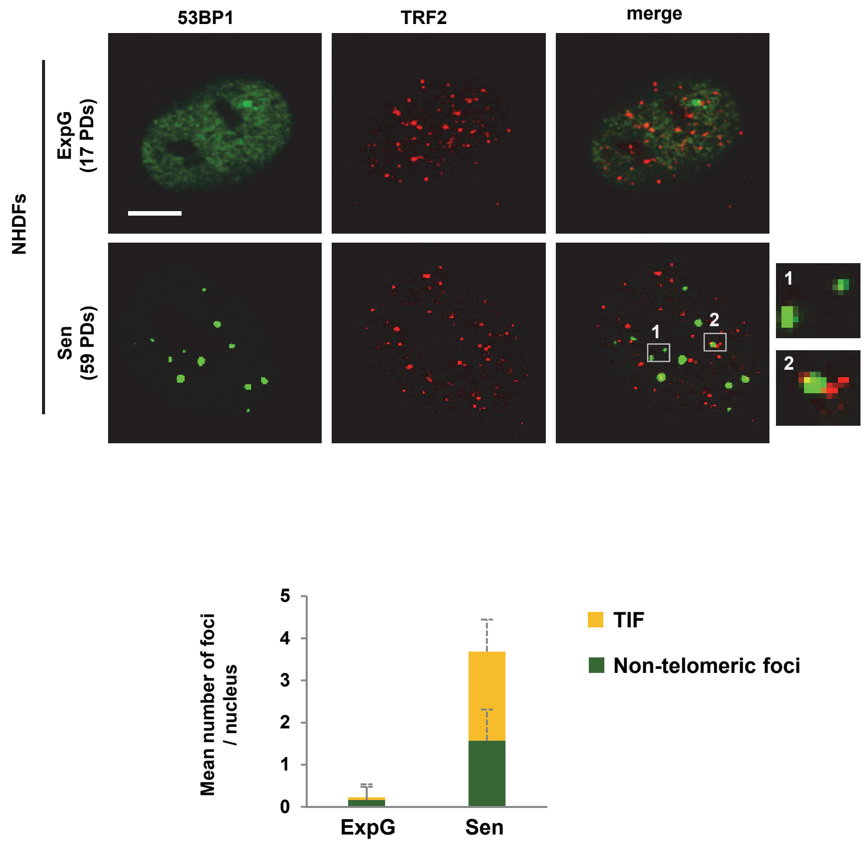

Supplementary Figure 5: DDR foci in senescent NHDFs are both telomeric and non telomeric (complements to Figure 2).

Upper panels: Representative confocal microscopy images for 53BP1 (green) and TRF2 (red) double immunofluorescence performed on exponentially growing and senescent NHDFs (donor 1MC). Lower panel: Foci double positive for 53BP1 and TRF2 (TIF) and foci positive only for 53BP1 (non-telomeric foci) were counted amongst at least 150 cells. The given results are the mean +/- SD of all counts.

ExpG=exponentially growing cells; Sen=cells at the senescence plateau. The exact PDs at which cells were taken is indicated.

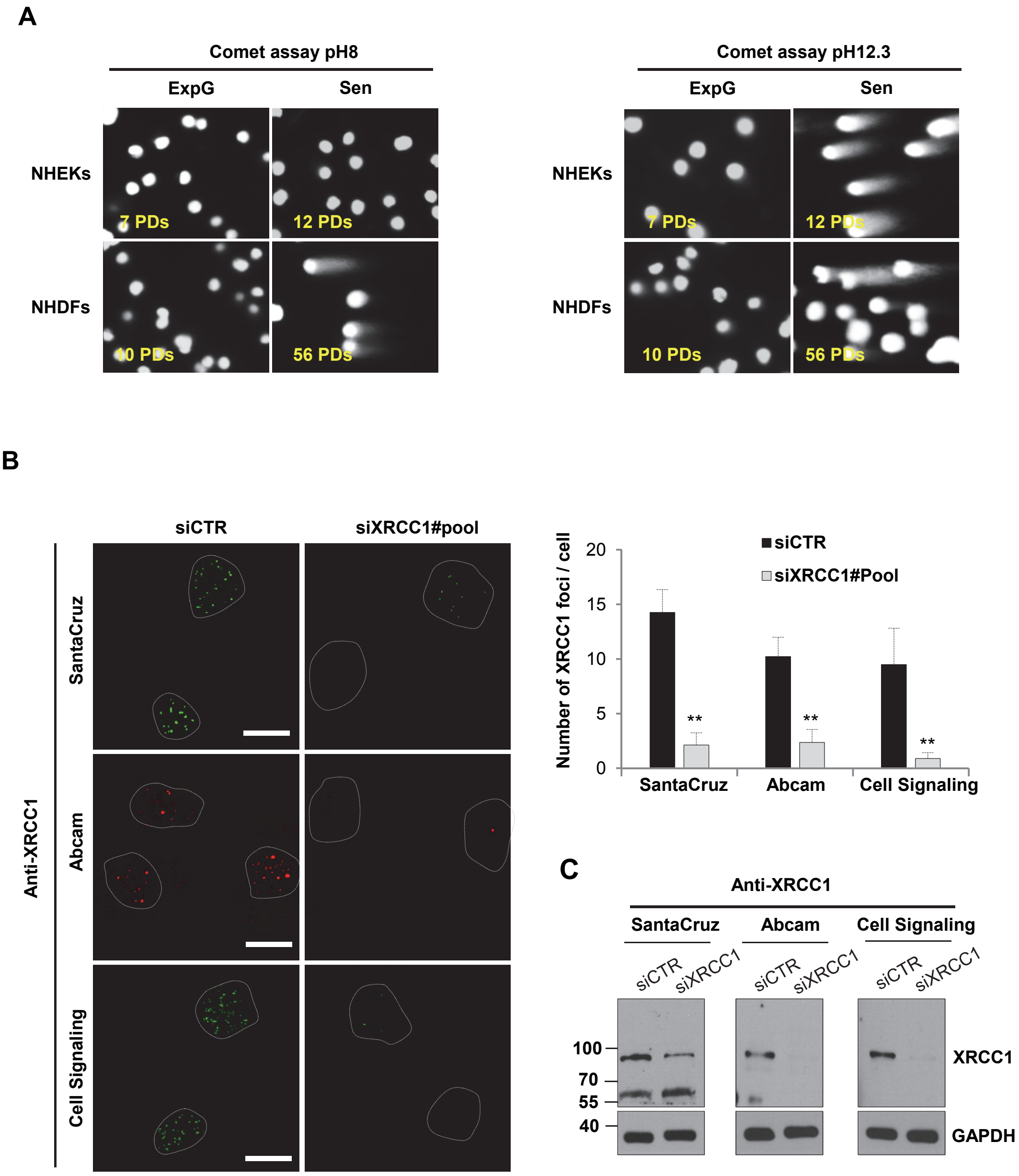

Supplementary Figure 6: complements to Figure 3.

(A) Representative ApoTome microscopy images of comet assays stained with SYBR ® Green whose quantitative analysis is given in Fig.3A. (B) Checking for the specificity of the XRCC1 antibodies. Three antibodies raised against three different immunogens were used: an antibody from Santa Cruz raised against the N-terminal 1-300 amino-acids, an antibody from Abcam raised against full length XRCC1, and an antibody from Cell Signaling raised against a peptide made of amino-acids around Arg300. Senescent NHEKs (donor 1MC) at 12.5 PDs transfected by a pool of control or anti-XRCC1 siRNAs were processed for immunofluorescence with the 3 antibodies. Left panel: Representative ApoTome microscopy images. Scale bar=10µm. Right panel: The number of XRCC1 foci per cell was counted in more than 40 cells. The bar chart represents the mean +/- SD of all counts (C) Analysis of the same cells as in B by western-blotting with the same 3 antibodies.

ExpG=exponentially growing cells; Sen=cells at the senescence plateau. The exact PDs at which cells were taken is indicated.

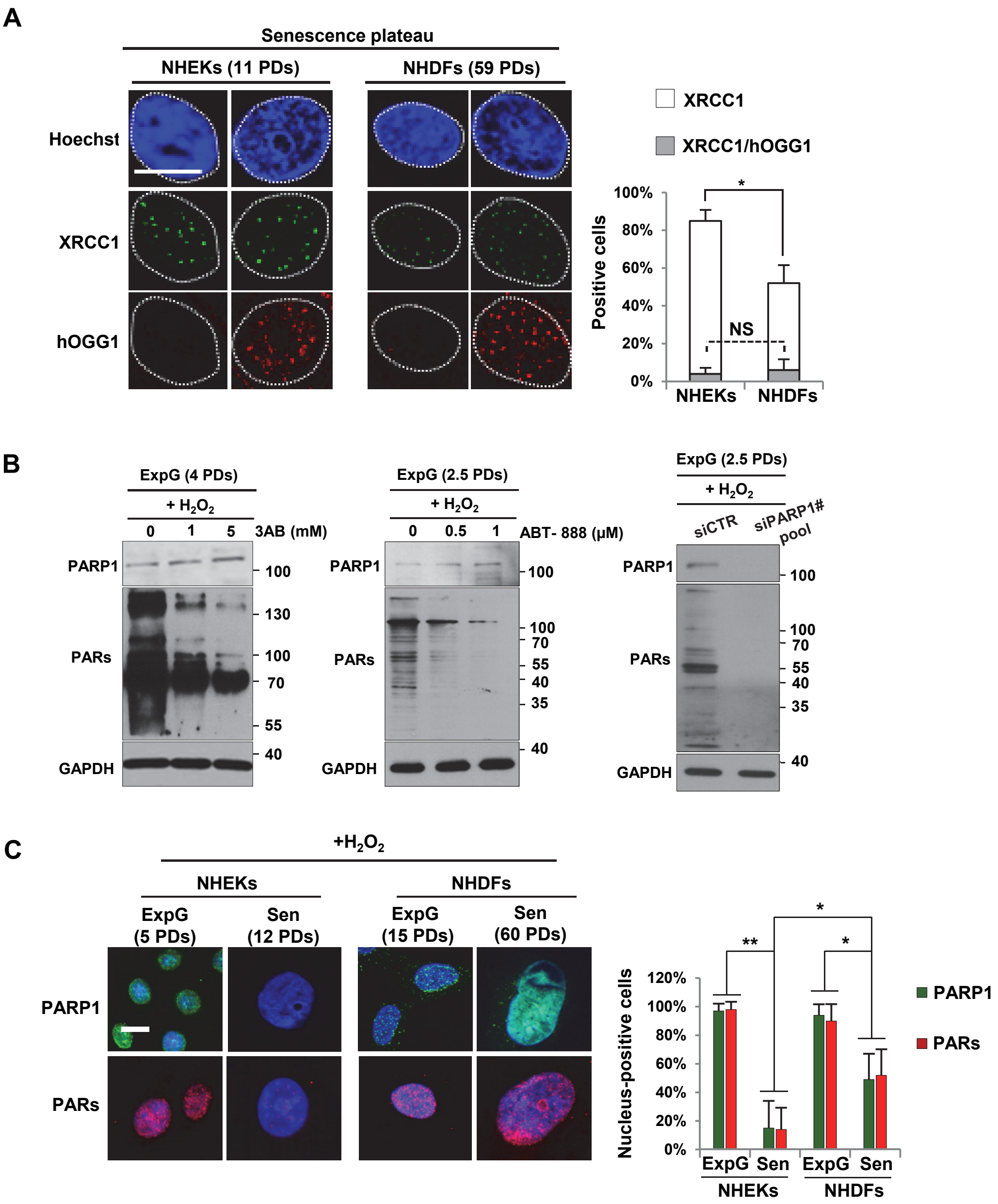

Supplementary Figure 7: complements to Figure 3

(A) XRCC1/hOGG1 double immunofluorescences performed on senescent NHEKs and NHDFs (donor 1MC). Left panel: Representative ApoTome microscopy images. Scale bar=10µm. Right panel: Positive cells were automatically quantified with ImageJ in 5 independent microscopic fields for a total of at least 100 cells for each case. The bar chart represents the means +/- SD of each 5 counts. (B) Checking for the specificity of anti-PARP1 and anti-PARs antibodies. Left and middle panels: western-blot analysis of PARP1, PARs and GAPDH (loading control) levels in total extracts of exponentially growing NHEKs (donor 1MC) treated with 3-AB or ABT888 during 24hrs, then with 100µM H_2O_2 at 4°C for 10min and then placed at 37°C for 5min. Right panel: western-blot analysis of PARP1, PARs and GAPDH (loading control) levels in exponentially growing NHEKs (donor 1MC) transfected with a pool of control or PARP1 siRNA. Four days after transfection, cells were treated by H_2O_2 as above. (C) PARP1 and PARs immunofluorescences performed on exponentially growing and senescent NHEKs and NHDFs (donor 1MC) treated as in Fig.3D. Left panel: Representative ApoTome microscopy images. Scale bar=10µm. Right panel: PARP1 and PARs positive cells were counted in 10 independent microscopic fields for a total of at least 100 cells for each case. The bar chart represents the means ± SD of each ten counts. The results are representative of 2 independent experiments.

ExpG=exponentially growing cells; Sen=cells at the senescence plateau. The exact PDs at which cells were taken is indicated.

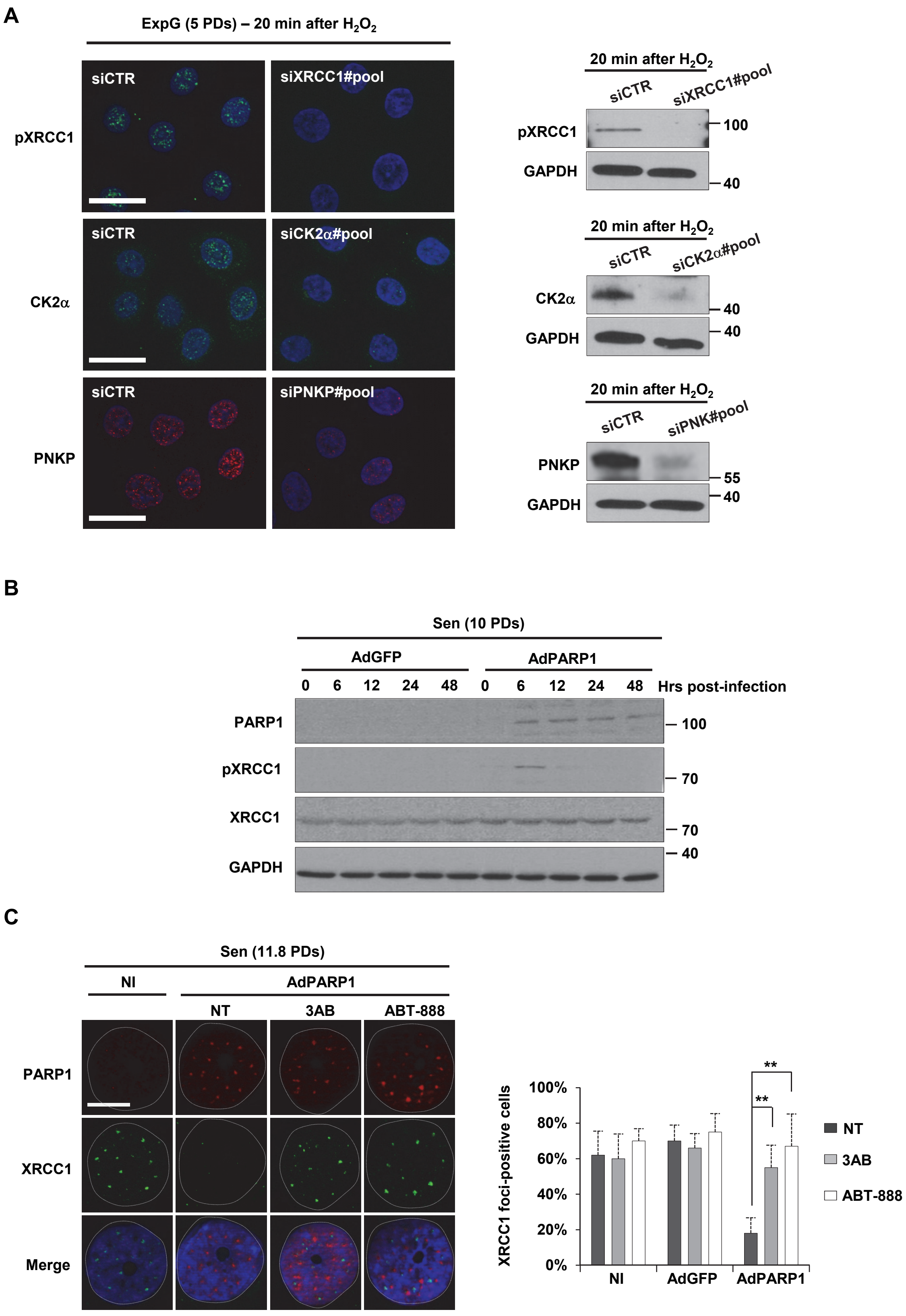
A
ExpG (5 PDs) – 20 min after H2O2
siCTR
siXRCC1#pool
pXRCC1
siCTR
siCK2α#pool
CK2α
siCTR
siPNKP#pool
PNKP
20 min after H2O2
siCTR
siXRCC1#pool
pXRCC1
GAPDH
100
40
20 min after H2O2
siCTR
siCK2α#pool
CK2α
GAPDH
40
40
20 min after H2O2
siCTR
siPNK#pool
PNKP
GAPDH
55
40
B
Sen (10 PDs)
AdGFP
AdPARP1
0 6 12 24 48 0 6 12 24 48 Hrs post-infection
PARP1
pXRCC1
XRCC1
GAPDH
100
70
70
40
C
Sen (11.8 PDs)
NI
AdPARP1
NT
3AB
ABT-888
PARP1
XRCC1
Merge
XRCC1 foci-positive cells
100%
80%
60%
40%
20%
0%
NI
AdGFP
AdPARP1
**
**
NT
3AB
ABT-888

Supplementary Figure 8: The PARP1 activity is necessary for the resolution of the XRCC1 foci (complements to Figure 4)

(A) Checking for the specificity of the pXRCC1, CK2α and PNKP antibodies. Exponentially growing NHEKs (donor 67FA1) were transfected with a pool of control, XRCC1, CK2α or PNKP siRNA. Two days after transfection, cells were treated by 100µM H_2O_2 at 4°C for 10min, placed at 37°C for 20min. Immunofluorescence and western-blot with the 3 antibodies were performed. Left: Representative ApoTome microscopy images . Scale bar = 20µm. Right: western-blot analysis.

(B) Senescent NHEKs (donor 67FA1) were infected with AdGFP or AdPARP1. Western-blot analysis of PARP1, phosphorylated XRCC1, total XRCC1 and GAPDH (loading control) at 0, 6, 12, 24 and 48hrs post-infection.

(C) Senescent NHEKs at 11.8 PDs (donor 67FA1) were infected with AdPARP1, AdGFP or kept non infected (NI) and treated or not with 5mM 3AB or 1µM ABT-888. Twenty four hours after infection, cells were processed for PARP1 and XRCC1 immunofluorescence. Left panel: representative photomicrographs. Scale bar=10µm. Right panel: Quantification of cells displaying XRCC1 foci. At least 100 cells were counted for each condition with image J. The bar chart represents the mean +/- SD. The results are representative of two independent experiments.

ExpG=exponentially growing cells; Sen=cells at the senescence plateau. The exact PDs at which cells were taken is indicated.

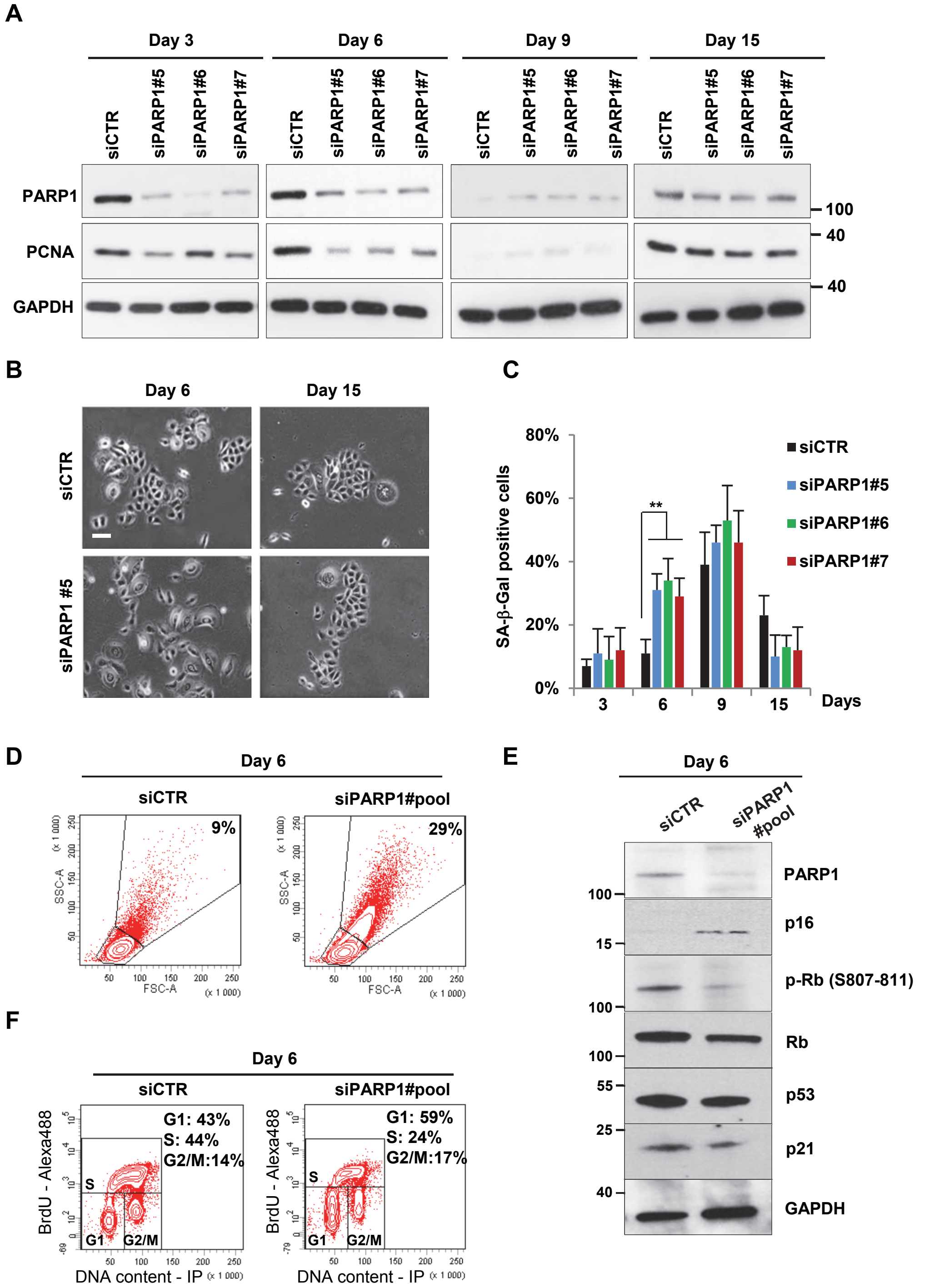

Supplementary Figure 9: complements to Figure 7.
(A) Western-blot analysis of PARP1, PCNA (proliferative index) and GAPDH (loading control) levels in total extracts of siCTR- and siPARP1-transfected NHEKs at the indicated time post-transfection. (B) Representative images of cell morphologies at days 6 and 15 post-transfection. Scale bar=50µm. (C) Percentage of SA-β-Gal-positive at days 3, 6, 9 and 15 post-transfection. The bar chart represents the means ± SD of each 4 counts. (D) Quantification of changes in cell morphology. siCTR- and siPARP1-transfected NHEKs were analyzed for size (FSC-A) and granularity (SSC-A) distribution by flow cytometry at day 6 post-transfection. (E) Western-blot analysis of PARP1, phosphorylated Rb (S807-811), Rb, p16, p53, p21, PCNA (proliferative index) and GAPDH (loading control) levels in total cell extracts. (F) Analysis by flow cytometry of the distribution of siCTR- and siPARP1-transfected NHEKs in G0/G1, S, and G2/M phases.

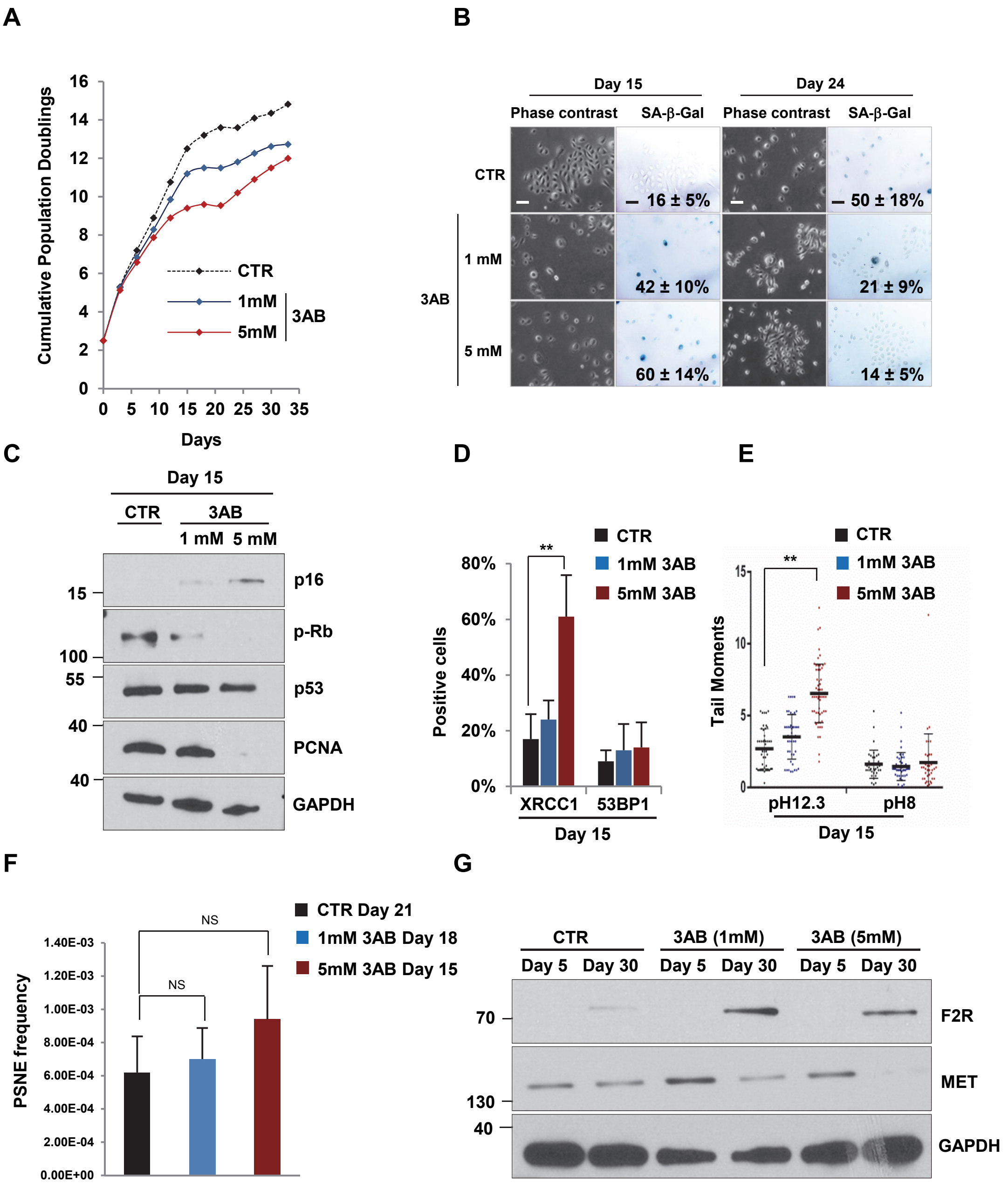

Supplementary Figure 10: Inhibiting PARP activity using 3AB induces premature senescence followed by PSNE

(A) Growth curve of NHEKs treated or not with 1 or 5mM 3AB every day. (B) Representative images of cell morphology and SA-β-Gal staining at the indicated time after the beginning of the treatment. The percentage of SA-β-Gal positive cells (means +/- SD) are given as inserts. (C) Western-blot analysis of p16, phosphorylated Rb, p53, PCNA (proliferation index) and GAPDH (loading control) 15 days after the beginning of the treatment. (D) Immunodetection of XRCC1 and 53BP1 foci 15 days after the beginning of the treatment. Positive cells were counted in 5 independent microscopic fields for a total of at least 50 cells for each case. The bar chart represents the means ± SD of each ten counts. (E) Alkaline (pH12.3) and neutral (pH8) comet assays performed in tandem 15 days after the beginning of the treatment. Tail moments of 30 to 50 comet-positive cells were quantified. Scatter dot plots represent the mean ± SD. (F) Measure of PSNE frequency of NHEKs as described in Materials and Methods. Counts of PSNE clones performed in 4 independent culture dishes. The given results are the mean +/- SD of all counts. (G) Western-blot analysis of the transformation markers F2R and MET and GAPDH as loading control in cells at the indicated time after the beginning of the treatment.

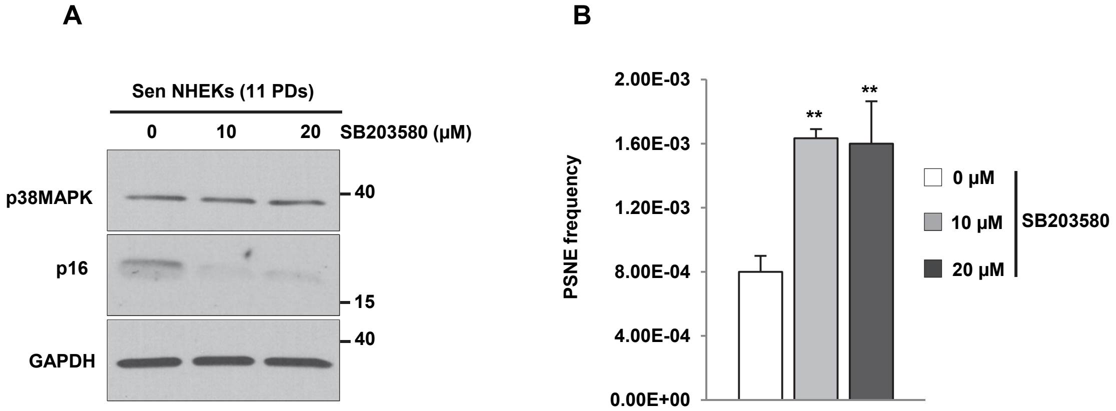

Supplementary Figure 11: Inhibiting the p38MAPK activity partially reverts the upregulation of p16 and increases the PSNE frequency

Senescent NHEKs at 11 PDs (donor 67FA1) were treated daily with 10 or 20 μM of SB203580. (A) Western-blot analysis of p16, p38MAPK and GAPDH (loading control) 24hrs after the beginning of the treatment. (B) Measure of PSNE frequency of NHEKs as described in Materials and Methods. Counts of PSNE clones performed in 3 independent culture dishes. The given results are the mean +/- SD of all counts.

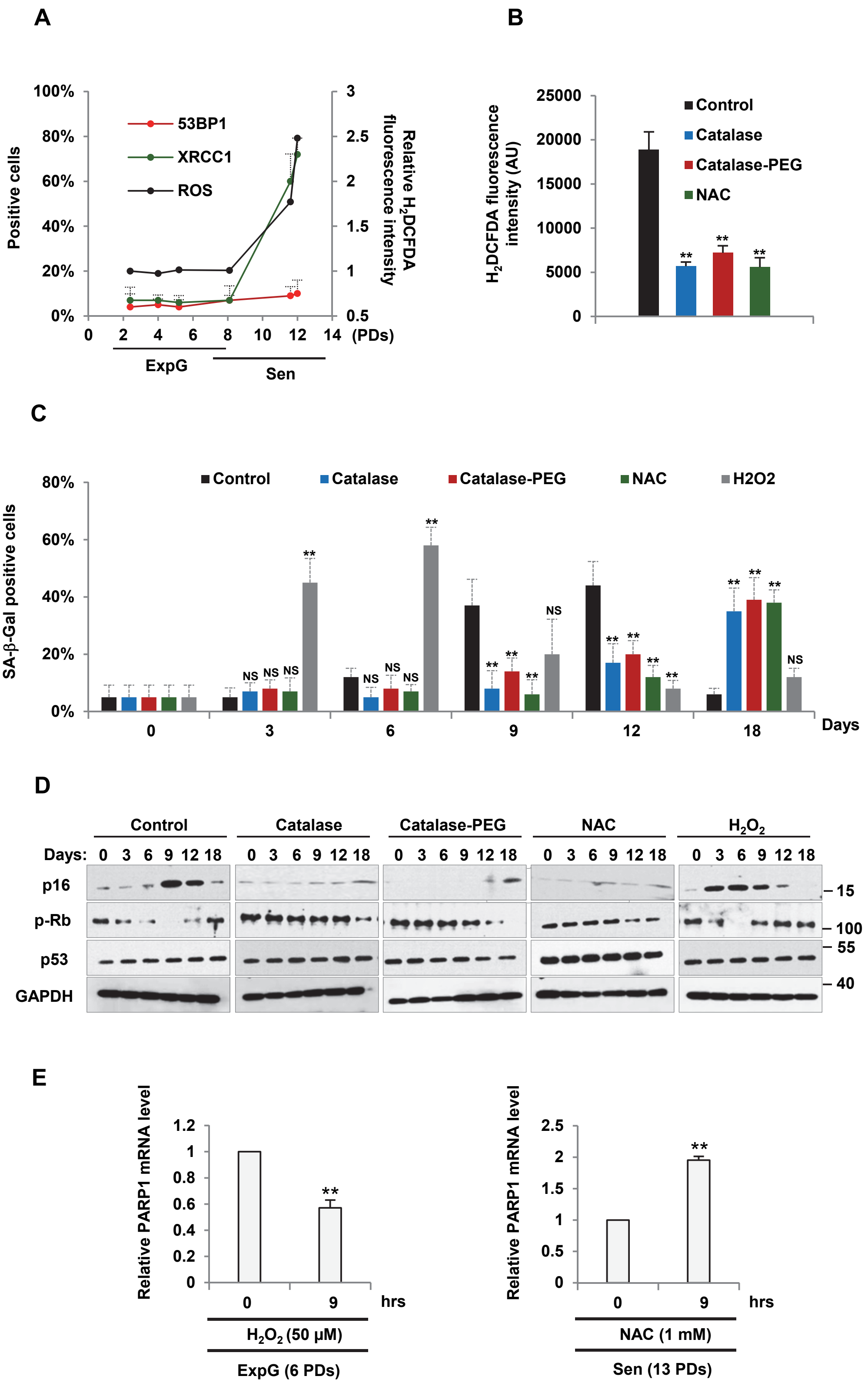
A
100%
80%
60%
40%
20%
0%
Positive cells
53BP1
XRCC1
ROS
3
2.5
2
1.5
1
0.5
Relative H2DCFDA fluorescence intensity
0 2 4 6 8 10 12 14 (PDs)
ExpG
Sen
B
25000
20000
15000
10000
5000
0
H2DCFDA fluorescence intensity (AU)
Control
Catalase
Catalase-PEG
NAC
**
C
80%
60%
40%
20%
0%
SA-β-Gal positive cells
Control
Catalase
Catalase-PEG
NAC
H2O2
NS
**
0 3 6 9 12 18
Days
D
Control
Catalase
Catalase-PEG
NAC
H2O2
Days: 0 3 6 9 12 18
p16
p-Rb
p53
GAPDH
15
100
55
40
E
Relative PARP1 mRNA level
1.2
1
0.8
0.6
0.4
0.2
0
0 9 hrs
H2O2 (50 μM)
ExpG (6 PDs)
2.5
2
1.5
1
0.5
0
NAC (1 mM)
Sen (13 PDs)

Supplementary Figure 12: Complements to Figure 8

(A) Immunofluorescence detection of 53BP1 and XRCC1 foci in NHEKs (donor 1MC) all along their cultivation, and, in parallel, measure of ROS concentration using H_2DCFDA as described in Materials and Methods. Positive cells were counted in 5 independent microscopic fields for a total of at least 100 cells for each case. Each point is the mean +/-SD of all counts (B) Verification of the efficacy of the anti-oxidant treatments performed in Fig.8. ROS concentration was measured 24hrs after the beginning of the treatment. The given results are means of triplicates ± SD. (C) Percentage of SA-β-Gal-positive cells (means ± SD) at days 0, 3, 6, 9, 12 and 18 of the experiment. (D) Western-blot analysis of p16, phosphorylated Rb, p53, PCNA (proliferative index) and GAPDH (loading control) levels in total cell extracts of treated and non-treated NHEKs at the indicated time of the experiment. (E) Expression of PARP1 is negatively regulated by oxidative stress. Exponentially growing or senescent NHEKs (donor 67FA1) were treated with 50µM H_2O_2 or 1mM of NAC respectively. RNA extractions were performed 9hrs post-treatment and PARP1 mRNA levels were analyzed by RT-qPCR. Results are means of triplicates ± SD. Data are representative of 2 independent experiments.

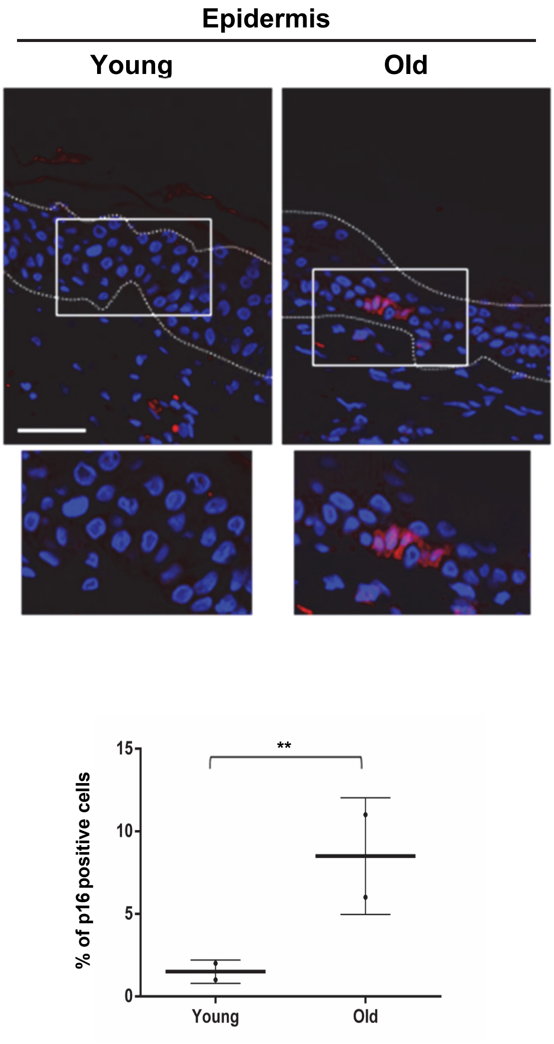

Supplementary Figure 13: Immunodetection of p16 in sections of skin from young versus old donors (complements to Figure 9)

P16 immunohistofluorescence performed in sections of skin samples from healthy human young (n=2) and old donors (n=2) (see sup table 2). Upper panels: Representative ApoTome microscopy images for epidermis of a young and an aged donor. Scale bar = 50µm. The square delimits the below image at higher magnification. Lower panels: Scatter dot plots indicating the percentage of positive epidermal cells in young and aged skin. Cells were counted in 10 independent microscopic fields for a total of at least 150 cells. The given results are the mean ± SD of the means in the 2 donors.

Fig 1F

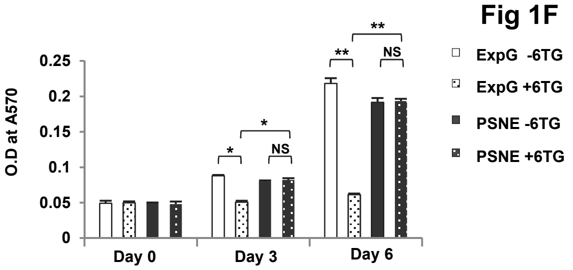

Fig 7G

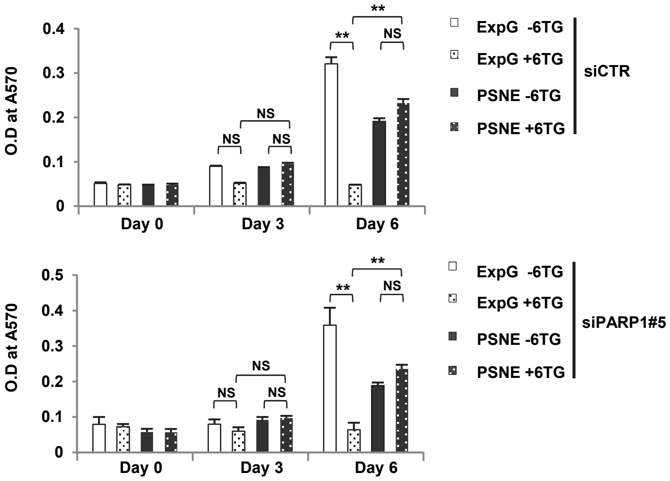

Fig 8I

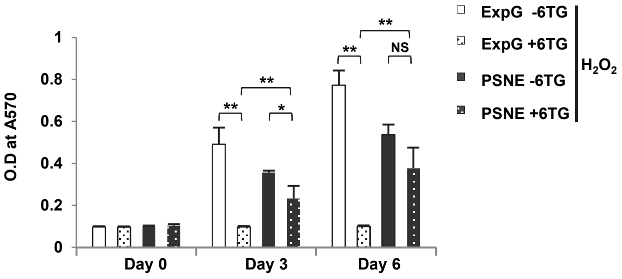

Fig 10E

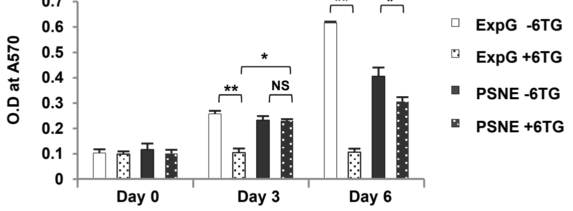

Supplementary Figure 14: Quantification of the hprt assays
The optic density of each crystal violet solution was measured three times. The bar charts represent the mean +/- SD of the three counts.

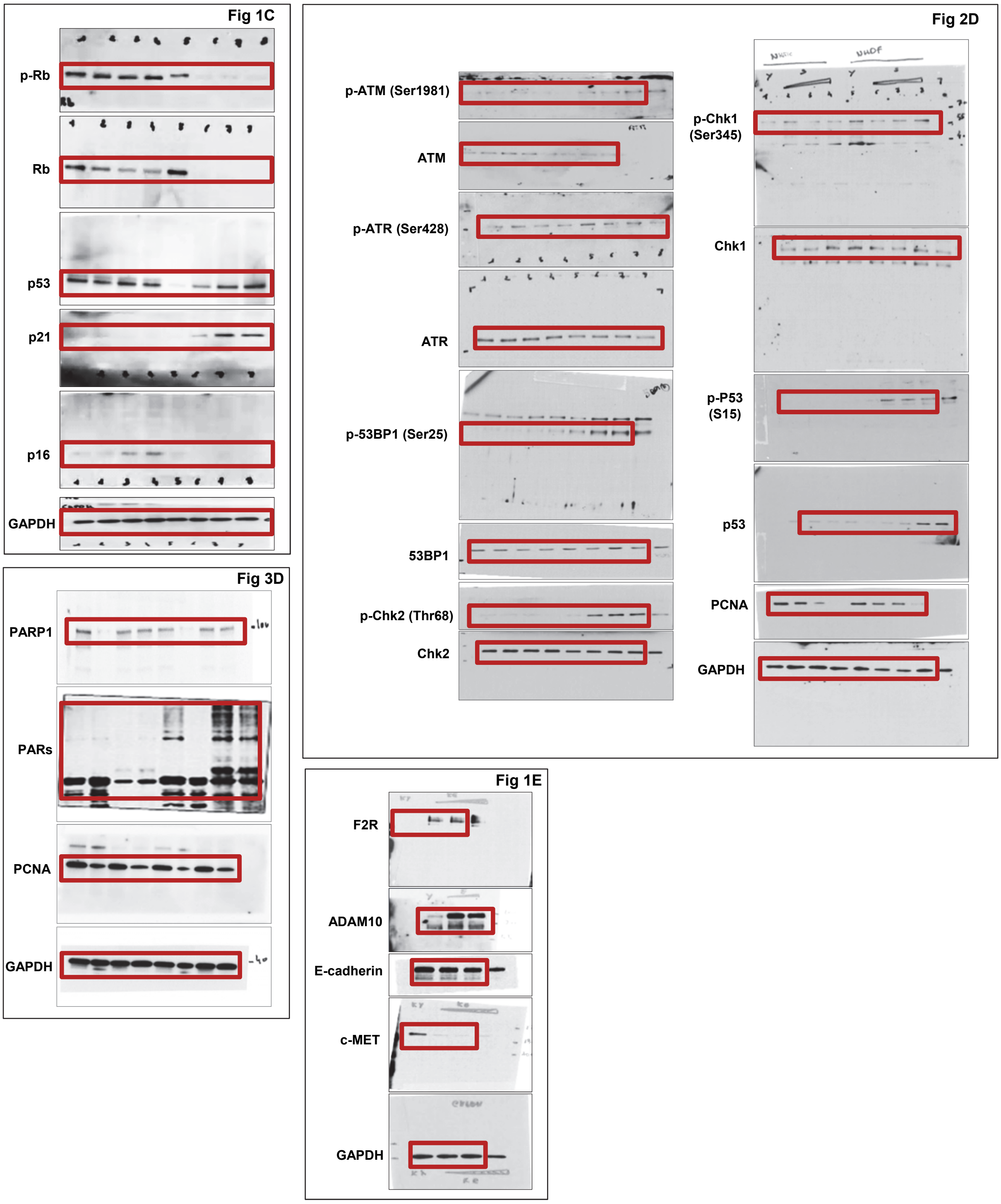

Supplementary Figure 15: Original scans of western blots

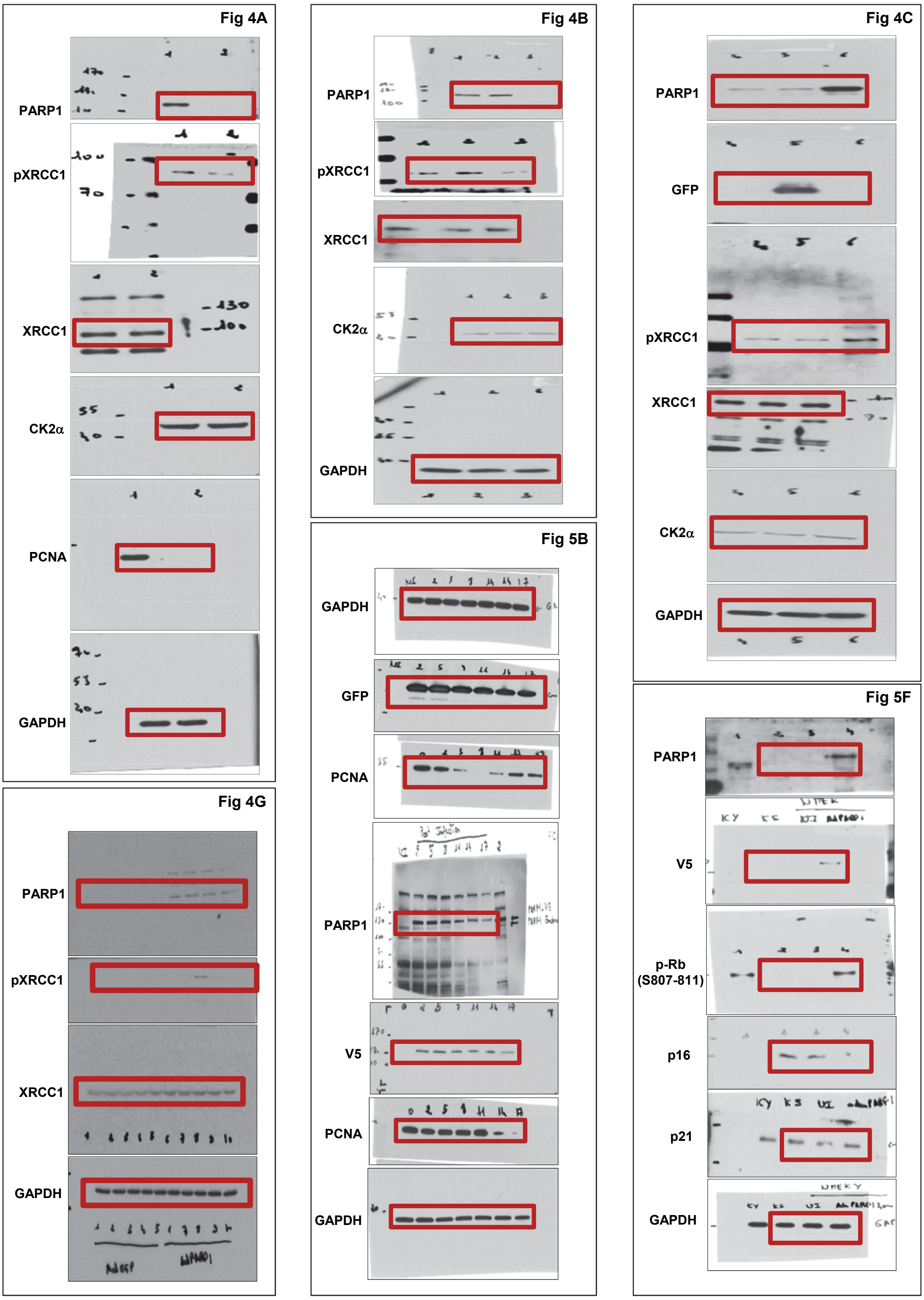

Supplementary Figure 16: Original scans of western blots

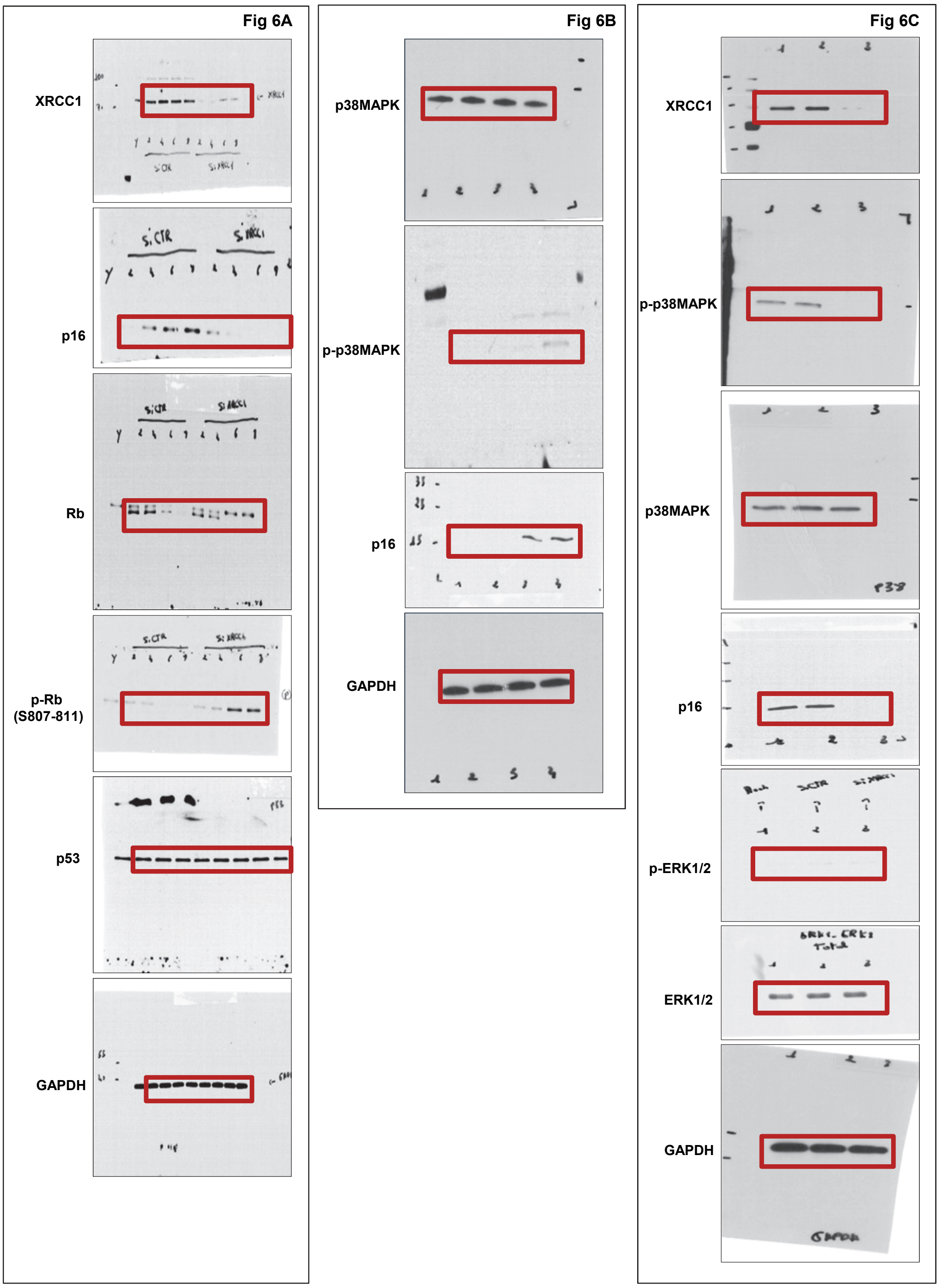

Supplementary Figure 17: Original scans of western blots

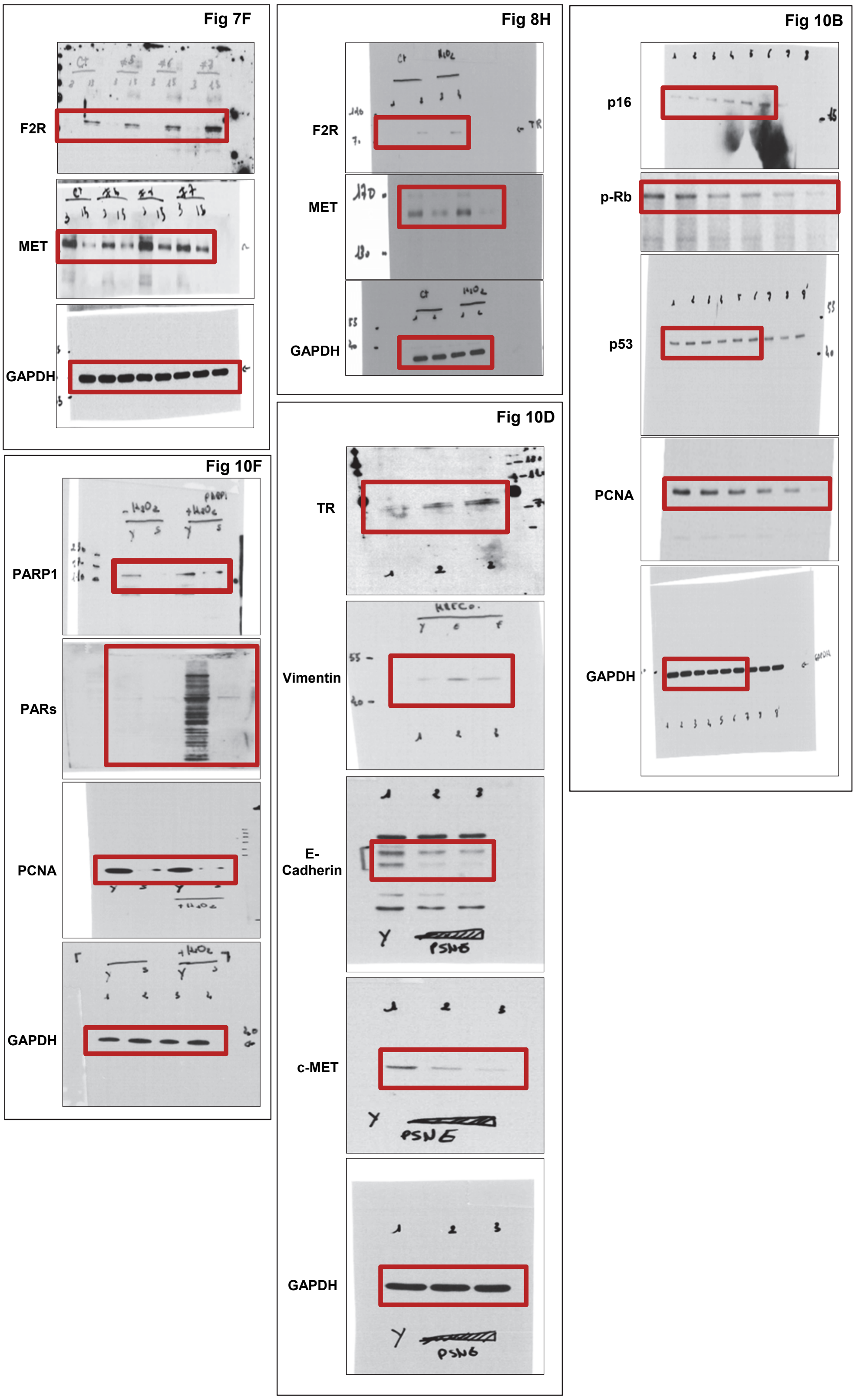

Supplementary Figure 18: Original scans of western blots

Supplementary Table 1: The different batches of primary cells used in the study

Donor	Company	Donor age / Race /Sex / location	Denomination	Cell type / Lot number	PDs at senescence plateau
1MC	Promocell	1 / Caucasian / male / foreskin	K1MC	Human epidermal keratinocytes / 6062703.3	11-12
			F1MC	Human dermal fibroblasts / 6062703.2	>55
1320	Tebu - bio	50 / Caucasian / female / facial skin	K1320	Human epidermal keratinocytes / 1320	10-11
			F1320	Human dermal fibroblasts / 1320	>38
67FA1	GIBCO – Invitrogen cell culture	67 / Asiatic / Female / breast skin	K67FA1	Human epidermal keratinocytes / 771287	13-15
			F67FA1	Human dermal fibroblasts / 771555	>38
2F19	Cambrex Bio Science	37 / Caucasian / Female / not communicated	K2F1958	Human epidermal keratinocytes / 2F1958	13-14
			F2F1966	Human dermal fibroblasts / 2F1966	>45
	Bio-Whittaker	58 / Black / Female	HMEC 0F1331	Human Mammary Epithelial Cells / 0F1331	10-11

Supplementary Table 2: The different samples of human skin used in the study

Skin biopsies from healthy "young" donors							
N° donor	Age	Sex	PARP1	XRCC1	53BP1	MnSOD	p16^{INK4a}
11391/09	29	male	x	x	x	x	x
32645/09	34	male	x	x	x	x	
35968/10	38	female	x	x	x		
33410/10	38	female				x	x
**Skin biopsies from healthy "old" donors**							
N° donor	Age	Sex	PARP1	XRCC1	53BP1	MnSOD	p16^{INK4a}
39853/10	65	male				x	x
9238/09	75	male	x	x	x		
17608/08	80	female	x	x	x		
12745/09	85	female	x	x	x	x	
28010/09	89	male	x	x	x	x	x

Supplementary Table 3: Primary antibodies used in this study

Antibodies	Compagny	Cat#	WB dilution	IF dilution	IHF dilution
PCNA	Dako	M0879	1:1000		
PCNA	Santa Cruz	sc-9857	1:500		
γH2AX	Novus Biologicals	NB100-78356	1:1000	1:100	
53BP1	Santa Cruz	sc-22760	1:500	1:200	1:50
53BP1	Novus Biologicals	NBP2-25028		1:100	
p-53BP1 (S25)	Bethyl Labrorateories	A300-652A	1:1000	1:100	
ATM	Santa Cruz	sc7230	1:500	1:100	
p-ATM (S1981)	Santa Cruz	sc-47739	1:500	1:100	
ATR	Santa Cruz	sc-1887	1:500	1:100	
p-ATR (S428)	Santa Cruz	sc-109912	1:500	1:100	
CHK2	Cell Signaling	3440	1:1000		
p-CHK2 (T68)	Cell Signaling	2661	1:1000		
CHK1	Santa Cruz	sc-8408	1:1000		
p-CHK1 (S345)	Abcam	ab47318	1:1000		
p53	Santa Cruz	sc126	1:1000	1:200	
p-p53 (S15)	Abcam	ab1431	1:1000	1:200	
p21	Santa Cruz	sc-6246	1:500		
p38MAPK	Cell Signaling	9218	1:1000		
p-p38MAPK (T180/T182)	Cell Signaling	9211	1:1000		
p16	Santa Cruz	sc1661	1:500		
p16	BD Pharmingen	550834	1:1000		1:50
Rb	Cell Signaling	9309	1:1000		
p-Rb (S807-811)	Cell Signaling	9308	1:1000		
PAR	Merck Chemicals	AM80, clone 10H	1:1000		
PARP1	Abcam	ab6079	1:400	1:100	
PARP1	Trevigen	4338-MC-50	1:1000	1:100	
XRCC1	Abcam	ab47920	1:1000	1:100	1:100
XRCC1	Cell Signaling	2735	1:1000	1:100	
XRCC1	Santa Cruz	sc-11429	1:500	1:200	1:50
p-XRCC1 (S518/T519/T523)	Bethyl Laboratories	A300-059A	1:1000	1:300	
CK2α	Abcam	ab137788	1:1000	1:100	
PNKP	Abcam	ab170954	1:1000	1:100	
DNA ligase3	GeneTex	GTX70147		1:100	
DNA ligase1	Medical and Biological Laboratories	K0190-3		1:100	
V5	Invitrogen	961-25	1:500		
GFP	Santa Cruz	sc-9996	1:500		
F2R	Santa Cruz	sc-13503	1:500		
MET	Invitrogen	37-0100	1:1000		
Vimentin	Santa Cruz	sc-6260	1:500		
Cadherin	Santa Cruz	sc-7870	1:500		
ADAM10	Santa Cruz	sc-48400	1:500		
GAPDH	Santa Cruz	sc-32233	1:1000		
MnSOD	Calbiochem	574596	1:1000		1:100
